# Goblet cells dictate viral tropism and pathogenesis in nasal and intestinal mucosae

**DOI:** 10.1073/pnas.2514150122

**Published:** 2025-10-08

**Authors:** Wenqian Wang, Wenwen Chao, Hui Zeng, Rongfeng Tang, Ruiling Liu, Chengcheng Wang, Xuan Wu, Jiaxin Qi, Yunlei Cao, Yuchen Li, Qian Yang

**Affiliations:** ^a^Ministry of Education Joint International Research Laboratory of Animal Health and Food Safety, Key Laboratory of Animal Physiology & Biochemistry, College of Veterinary Medicine, Nanjing Agricultural University, Nanjing, Jiangsu 210095, China

**Keywords:** nasal and intestinal mucosae, goblet cell, porcine epidemic diarrhea virus, swine influenza virus, goblet cell associated antigen passages

## Abstract

Host determinants are critical for shaping the outcomes of viral mucosal infections and developing effective antiviral strategies. However, the regulatory roles of “nonsusceptible” mucosal cells remain unclear. Here, we show that while swine influenza and porcine epidemic diarrhea (PEDV) viruses infect piglet nasal and intestinal epithelia, each virus establishes efficient infection only in its preferred mucosal niche. Goblet cell activity significantly influences mucosal infection outcomes; increased mucus secretion effectively blocks viral entry, while its reduction facilitates viral dissemination. Notably, PEDV activates acetylcholine-cholinergic receptor muscarinic 3 signaling in submucosal enteric neurons to induce goblet cell-associated antigen passages. This mechanism enables the translocation of intestinal bacteria to the lamina propria in early infection, even when the epithelial barrier remains intact, triggering inflammation and exacerbating mucosal damage. Our findings emphasize the crucial role of goblet cells in controlling viral tropism and their potential as targets for developing effective broad-spectrum antiviral approaches.

## Significance

We identify goblet cells as regulators of viral infections at mucosal surfaces. Goblet cell-derived mucus effectively shields nasal and intestinal epithelia from swine influenza virus (SIV) and porcine epidemic diarrhea virus (PEDV), with virus-driven modulation of mucus secretion determining infection containment or dissemination. Remarkably, PEDV not only suppresses mucus secretion but also rapidly induces goblet cell-associated antigen passages (GAPs) through an acetylcholine–cholinergic receptor muscarinic 3 neuro-epithelial axis. GAPs enable luminal bacteria to cross the intact epithelial barrier during the initial infection phase, triggering an acute inflammatory cascade that significantly exacerbates mucosal damage. Our findings reveal goblet cells and their neuronal signaling circuits as key determinants of viral mucosal pathogenesis and promising targets for developing broad-spectrum antiviral interventions.

Mucosal surfaces of the gastrointestinal, respiratory, and urogenital tracts form the body’s primary interface with the external environment and are the main portals of entry for most viruses (1). Understanding the host determinants that govern viral behavior at mucosal barriers is essential for curbing viral colonization and transmission, as well as for improving therapeutic interventions. Typically, infection starts when virions bind epithelial entry receptors and are activated by host proteases, enabling fusion, entry, and replication (2). The distribution of these receptors and proteases is often viewed as a key determinant of viral tropism, yet their expression extends beyond tissues that support productive replication. For instance, angiotensin-converting enzyme 2 and insulin like growth factor 1 receptor, the receptors for Severe Acute Respiratory Syndrome Coronavirus 2 (SARS-CoV-2) and respiratory syncytial virus (RSV), are widely distributed, yet both viruses replicate mainly in the respiratory epithelium (3, 4). Likewise, influenza viruses depend on sialic acid receptors for attachment and on TMPRSS2 for hemagglutinin activation (5), whereas flaviviruses such as dengue, West Nile, and Zika require furin-mediated maturation (6). However, despite the widespread expression of these receptors and proteases across diverse epithelial and stromal tissues, replication of these viruses remains largely restricted to defined mucosal or tissue niches. In addition to these factors, mucosal immune states play a decisive role in shaping infection outcomes. Nonetheless, the intensity of mucosal immune responses does not always correlate with infection efficiency, largely because of viral immune evasion strategies, from suppressing interferon pathways and innate immune cell function to infecting and manipulating immune cells for dissemination (7, 8). Consequently, although numerous host factors related to viral mucosal infections have been identified, the core mechanisms by which viruses establish infections at mucosal sites have not been fully elucidated.

Viral entry, replication, and dissemination in mucosal tissues involve complex, multilayered interactions among diverse cell types rather than a simple one-way process between “susceptible” cells and viruses. Evidence increasingly shows that “nonsusceptible” mucosal cells also play pivotal roles in regulating infection and pathogenesis. For example, norovirus and rotavirus target tuft and intestinal epithelial cells, respectively, but require M cells to establish efficient intestinal infection (9). Similarly, although RSV primarily infects alveolar epithelial cells, its lung pathology is largely driven by aberrant neutrophil infiltration (10). Such findings underscore the critical influence of nonsusceptible cells in viral infection outcomes and highlight the necessity of looking beyond susceptible cells to fully elucidate mucosal infection dynamics. However, conventional bulk omics average signals across heterogeneous cell populations, obscuring the transcriptional and regulatory heterogeneity that underlies mucosal viral pathogenesis. Single-cell transcriptomics overcomes this limitation by resolving rare but functionally critical cell populations and capturing their dynamic roles during infection, thereby enabling fine-scale dissection of the infection process (11). Using this approach, researchers have identified nonsusceptible cell subsets in influenza and hepatitis B virus models that critically influence infection outcomes (12, 13).

The swine influenza virus (SIV) typically initiates infection in the nasal mucosa before disseminating to the lower respiratory tract, but mounting evidence indicates that it can also infect the intestinal tract (14, 15). Similarly, porcine epidemic diarrhea virus (PEDV) primarily targets the small-intestinal mucosa yet is capable of invading the nasal mucosa, resulting in enteric disease in piglets (16). This potential “cross-mucosal infection” mechanism of both pathogens offers a valuable opportunity to explore mucosal tropism across tissues and identify underlying host determinants. Here, we used in vivo and ex vivo models to elucidate how SIV and PEDV manipulate goblet cell function to establish and sustain mucosal infections. From single-cell sequencing of respiratory and intestinal mucosae, we found that goblet cell remodeling strongly correlates with local infection outcomes and further underscoring its pivotal role in the mucosal tropism of both viruses. Collectively, these findings refine the understanding of SIV and PEDV pathogenesis at mucosal surfaces and lay a foundation for developing targeted mucosal antiviral strategies.

## Results

### Differential Infection Outcomes of PEDV and SIV in Piglet Nasal Mucosa.

**Fig. 1.**
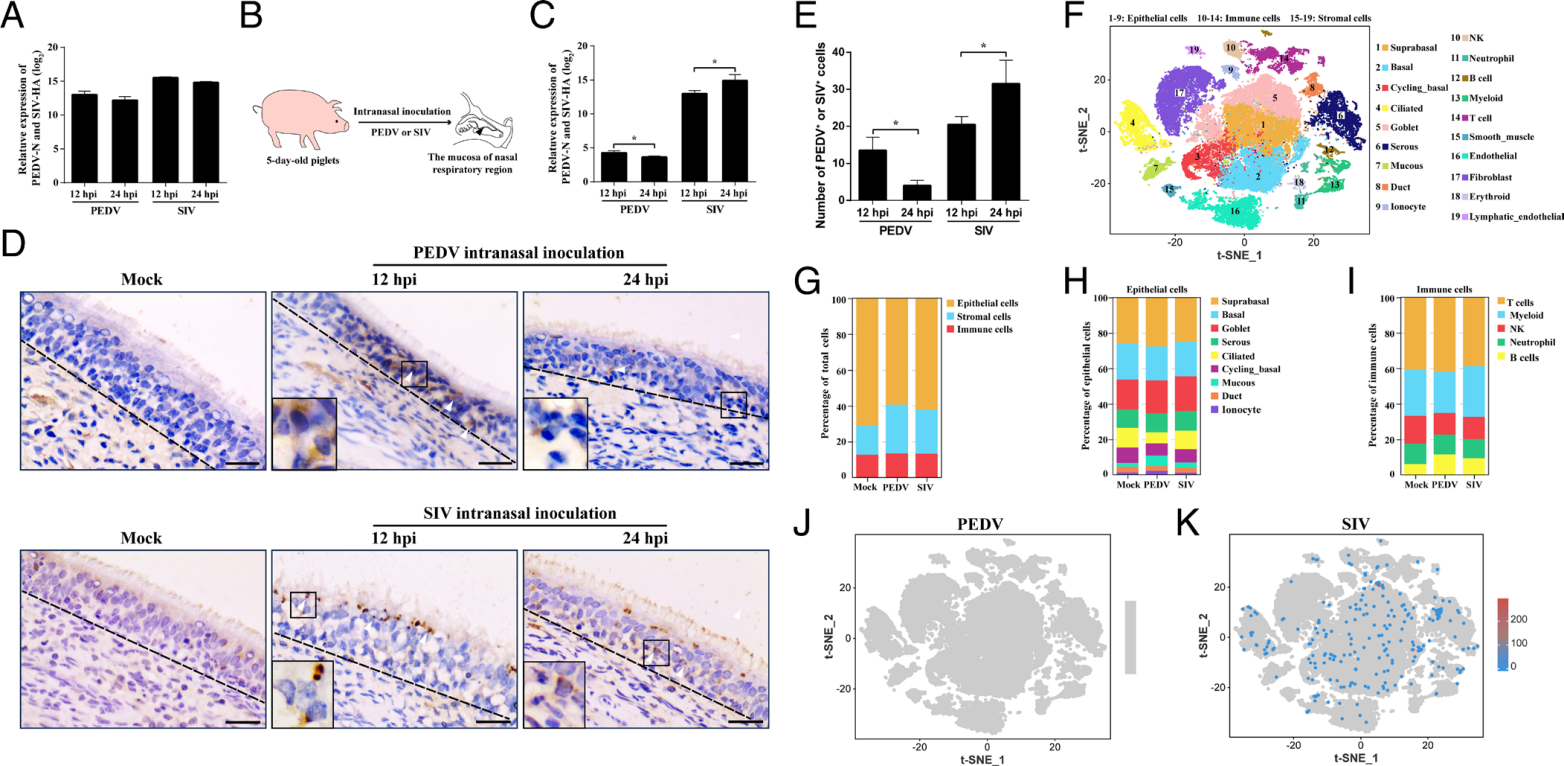
Differential infection outcomes of PEDV and SIV in piglet nasal mucosa. (*A*) Relative messenger RNA (mRNA) levels of PEDV-N and SIV-HA in NECs at 12 and 24 hpi. (*B*) Schematic of the intranasal inoculation model in 5-d-old piglets. (*C*) Viral gene expression dynamics in nasal mucosa postinoculation. (*D*) IHC showing PEDV and SIV distribution at 12 and 24 hpi; (Scale bar, 20 μm.) (*E*) Quantification of PEDV^+^ or SIV^+^ epithelial cells (three sections per piglet, three random fields per section). (*F*) t-distributed stochastic neighbor embedding (t-SNE) projection of nasal mucosal cells annotated by major cell types. (*G*–*I*) Proportions of total cell types (*G*), epithelial subsets (*H*), and immune subsets (*I*) in mock, PEDV-, and SIV-infected groups. (*J* and *K*) t-SNE maps showing PEDV (*J*) or SIV (*K*) distribution across cell subsets. Data are mean ± SD of three independent experiments; *P < 0.05.

We established an air–liquid interface culture model of porcine nasal epithelial cells (NECs) to characterize the infection properties of PEDV and SIV. Quantitative reverse transcription PCR analysis showed that both viruses successfully infected piglet NECs and displayed similar postinfection replication kinetics (Fig. 1*A*). In a piglet nasal aerosol infection model (Fig. 1*B*), RT-qPCR showed that PEDV titers peaked at 12 h postinfection (hpi) and then declined sharply, indicating a short-lived replication phase, whereas SIV RNA levels continued to rise through 24 hpi and remained high (Fig. 1*C*). Immunohistochemistry (IHC) revealed corresponding spatial patterns: PEDV-positive epithelial cells were sparse at 12 hpi and markedly reduced by 24 hpi, while SIV formed dense foci at 12 hpi that expanded within the epithelium and breached the basement membrane to invade the lamina propria by 24 hpi (Fig. 1 *D* and *E*). Hence, although both viruses infect NECs, PEDV causes transient localized infection, whereas SIV efficiently penetrates and spreads across the mucosa.

To investigate the mechanisms underlying these differences, we performed single-cell RNA sequencing (scRNA-seq) of piglet nasal mucosa at 24 hpi. Based on cell-specific marker genes, nasal mucosal cells were grouped into epithelial, immune, and stromal categories and further subdivided into 19 cell subpopulations (Fig. 1*F*). Epithelial populations showed the largest infection-associated changes, with distinct virus-specific patterns: PEDV reduced cycling basal and ciliated cells but increased mucous cells, while SIV reduced cycling basal cells without affecting ciliated or mucous cells (Fig. 1 *G* and *H*). Immune cell composition remained stable in both groups (Fig. 1*I*). Viral RNA mapping revealed no PEDV reads in any cell subpopulation at 24 hpi, indicating failure to establish infection (Fig. 1*J*). In contrast, SIV RNA was detected in multiple epithelial subtypes, including cycling basal, goblet, and ciliated cells (Fig. 1*K* and *SI Appendix*, Fig. S1*A*), consistent with its sustained replication and expansion in the nasal mucosa.

### Virus Receptors, Host Proteases, and Mucosal Immunity Do Not Correlate with the Differential Tropism of PEDV and SIV in Nasal Mucosa.

Transcriptional profiles of viral entry receptors and host proteases in NECs were analyzed to elucidate the molecular basis of differential nasal mucosal tropism of PEDV and SIV. The results indicated consistently high baseline expression of essential host proteases (TMPRSS2, FURIN), shared receptors/coreceptors (Epidermal Growth Factor Receptor (EGFR), TFR1) (17, 18), PEDV-associated entry factors (OCLN, Integrin β3) (19), and SIV-associated entry molecules (mGluR2, UVRAG, Cav1.2) (20–22) across epithelial subpopulations, with no obvious differences between virus-infected and mock groups (*SI Appendix*, Fig. S1*B*). Moreover, these receptors and proteases exhibited similar expression patterns across nasal epithelial subpopulations (*SI Appendix*, Fig. S1*C*). These findings suggest that the abundance and distribution of viral entry-associated receptors and host proteases in NECs are unlikely to explain the differences in nasal mucosal tropism between PEDV and SIV.

The epithelial and immune cells collaboratively mediate innate and adaptive immunity, effectively establishing the immune barrier of the nasal mucosa. We therefore compared transcriptional profiles of immune-related genes in both cell types after PEDV and SIV infections to determine whether differential immune responses explain the distinct mucosal infection patterns observed. Kyoto Encyclopedia of Genes and Genomes (KEGG) analysis showed that both viruses activated similar infectious disease–related and innate immune pathways, including Toll-like, NOD-like, and Retinoic Acid-Inducible Gene (RIG)-I-like receptor signaling, and upregulated canonical antiviral genes such as GBP1, GBP2, MX1, MX2, STAT1, and STAT2 (*SI Appendix*, Fig. S2 *A*–*D*). Although stronger immune responses are often linked to faster viral clearance, SIV induced robust immune activation persisted, while PEDV triggered weaker responses but caused only transient infection, indicating that immune response magnitude alone does not explain their divergent outcomes.

Among immune cell subsets, T cells (40.8%) and myeloid cells (25.9%) comprised over 60% of the nasal immune compartment and mirrored the overall immune transcriptional profile in both infections. These subsets were enriched in RIG-I-like and NOD-like receptor signaling and infectious disease pathways (e.g., influenza A, measles) and showed similar innate immune gene expression changes (*SI Appendix*, Fig. S2 *E*–*H*), suggesting they are key effector populations in mucosal immunity to PEDV and SIV.

### Distinct Transcriptional Changes in Nasal Goblet Cells Are Closely Associated with the Differential Infection Outcomes of PEDV and SIV.

Although immune-related gene profiles in NECs did not strongly correlate with the distinct mucosal infection outcomes of PEDV and SIV, both viruses altered epithelial cell number, composition, and global transcriptional patterns in a virus-specific manner (*SI Appendix*, Fig. S3*A*). Given the functional heterogeneity of nine epithelial subpopulations, we analyzed cell–cell interactions and transcriptional profiles separately. PEDV infection significantly enhanced interactions among cycling basal, ciliated, and goblet cells and with other epithelial subsets, whereas SIV markedly reduced such interactions (*SI Appendix*, Fig. S3*B*). Differential expression analysis confirmed distinct virus-specific changes in these cell types (*SI Appendix*, Fig. S3*C*).

Cycling basal cells, essential for epithelial repair, and ciliated cells, key for mucociliary clearance and often primary viral targets (23), showed activation of innate immune and infectious disease pathways (e.g., RIG-I-like receptor signaling, influenza A) and upregulation of antiviral genes (STAT1, ISG15, MX2) in both infections (*SI Appendix*, Fig. S3 *D*–*G*). Notably, PEDV, despite failing to establish efficient infection, induced stronger transcriptional modulation than SIV, repressing genes for proliferation (TOP2A, PCNA), tight junctions (TJP1, TJP3), and apoptosis (CASP3, FADD), while activating ciliogenesis genes (FOXJ1, DNAH3) (*SI Appendix*, Fig. S3 *H* and *I*). However, these changes did not align with divergent infection outcomes, suggesting these cells are not the main drivers of differential tropism.

Goblet cells secrete mucus, forming a crucial barrier against pathogens in the nasal mucosa (23). Our analyses showed that PEDV infection significantly activated multiple pathways regulating mucus secretion, including protein processing in the endoplasmic reticulum and protein export (*SI Appendix*, Fig. S3*J*), accompanied by the upregulation of mucus antiviral molecules and mucus secretion-associated genes (such as MUC5AC, TFF3, XBP1, and KLF4) (*SI Appendix*, Fig. S3*K*). Conversely, SIV infection inhibited mucus secretion-related signaling pathways in goblet cells, including reactive oxygen, Nuclear Factor-κB (NF-κB), and PI3K-Akt (*SI Appendix*, Fig. S3*J*), and it suppressed the transcription of mucus antiviral molecules (such as MUC5AC and TFF3; *SI Appendix*, Fig. S3*K*). These opposing effects indicate that PEDV may promote mucus secretion to limit its persistence, while SIV suppresses mucus production to sustain infection, potentially shaping their distinct nasal mucosal tropisms.

### PEDV and SIV Nasal Mucosal Tropism Is Associated with the Mucus Secretion Function of Nasal Goblet Cells.

**Fig. 2.**
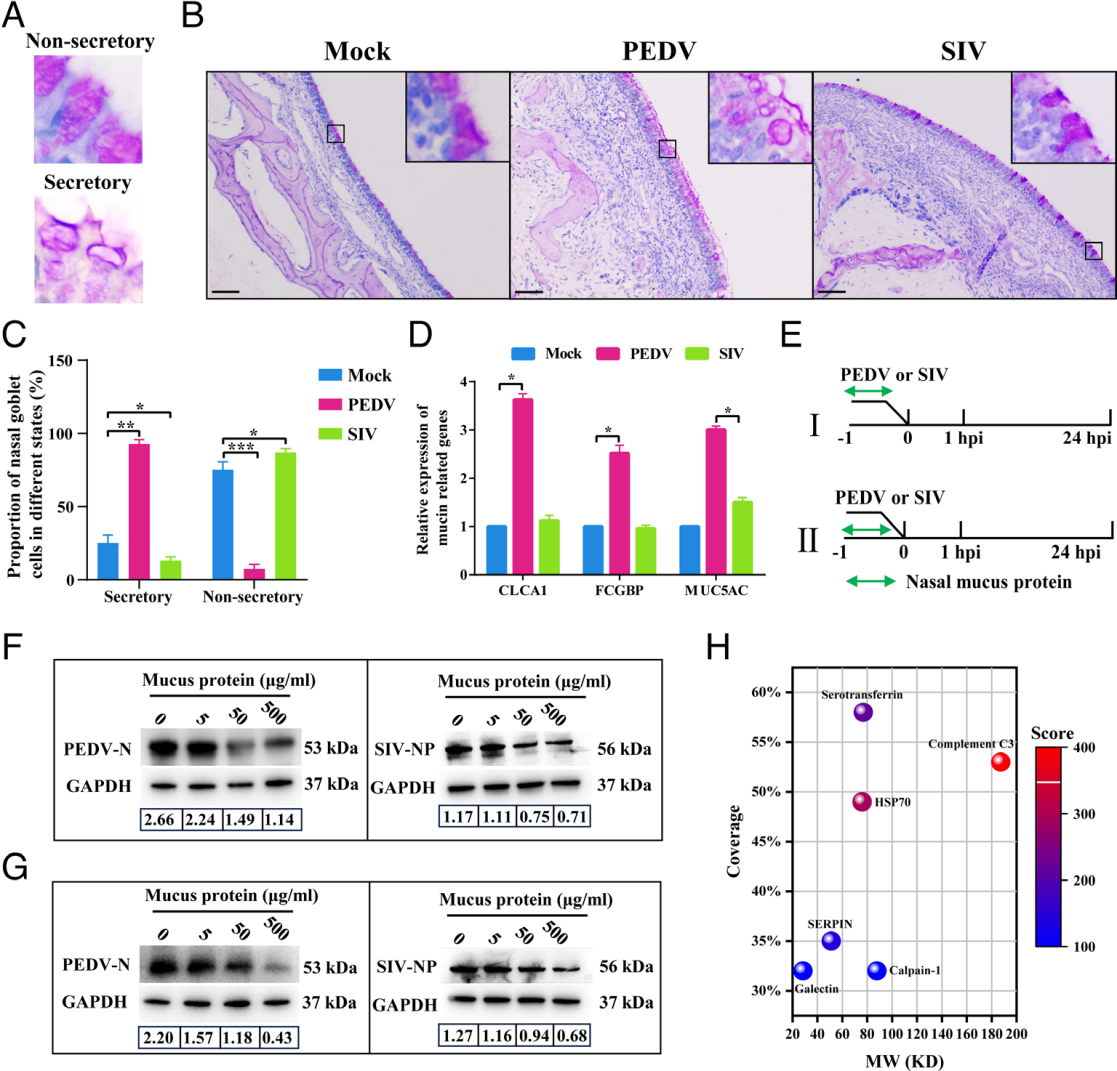
PEDV and SIV nasal mucosal tropism is associated with the mucus secretion function of nasal goblet cells. (*A*) Morphological features of secretory and nonsecretory nasal goblet cells in piglets. (*B*) Representative PAS-stained nasal mucosa images from mock-, PEDV-, and SIV-infected piglets; (Scale bar, 20 μm.) (*C*) Quantification of secretory and nonsecretory goblet cell proportions among total goblet cells in panel *B* (four random fields per section, three sections per pig). (*D*) Relative expression levels of mucin-related genes in nasal mucosa across groups. (*E*) Experimental design for determining the stage at which total nasal mucus protein inhibits PEDV or SIV: I, virus pretreatment; II, cell pretreatment. (*F* and *G*) Western blot analysis of the antiviral effects of nasal mucus protein after virus pretreatment (*F*) or cell pretreatment (*G*); numbers indicate grayscale ratios of viral protein to GAPDH. (*H*) Liquid Chromatography–Tandem Mass Spectrometry (LC–MS/MS) analysis of nasal mucus proteins; bubble plot shows protein identity, coverage (*y*-axis), molecular weight (*x*-axis), and score (color). Data are mean ± SD from three independent experiments; *P < 0.05, **P < 0.01, ***P < 0.001.

Based on single-cell sequencing data, we observed differences in nasal goblet cell responses to PEDV or SIV infections. To validate these findings, we evaluated the mucus secretion by infected nasal goblet cells using Periodic acid–Schiff (PAS). Under physiological conditions, goblet cells can be categorized as “secretory” or “nonsecretory” depending on mucin accumulation or release (Fig. 2*A*). Our results showed that PEDV infection significantly increased the proportion of secretory goblet cells by approximately 68% and markedly thickened the nasal mucus layer, whereas SIV infection reduced this proportion by approximately 12% (Fig. 2 *B* and *C*). To further validate the PAS staining results, we performed immunofluorescence staining for the goblet cell-specific mucin protein MUC5AC. Immunofluorescence analyses showed that PEDV infection markedly increased MUC5AC release onto the epithelial surface, accompanied by significantly reduced intracellular MUC5AC fluorescence in goblet cells. Conversely, SIV infection resulted in substantial intracellular accumulation of MUC5AC, indicative of impaired mucin secretion (*SI Appendix*, Fig. S4 *A* and *B*). RT-qPCR showed that PEDV significantly upregulated the transcription of key mucin-related genes (CLCA1, MUC5AC, and FCGBP), with CLCA1 showing the greatest increase. Conversely, SIV infection had no significant effect on these genes (Fig. 2*D*), suggesting that PEDV promoted mucus secretion, whereas SIV inhibited it.

Although mucus is essential for respiratory health, direct evidence of its antiviral function in porcine nasal tissues remains limited. To address this gap, we extracted total protein from piglet nasal mucus and pretreated the viral and host cells separately (Fig. 2*E*). Nasal mucus proteins significantly inhibited PEDV or SIV infections, irrespective of whether the treatment targeted the virus or host cells (Fig. 2 *F* and *G*). Proteomic analysis revealed that nasal mucus was enriched with several molecules with potential antiviral activity, including serine protease inhibitors, galectins, calpain-1, heat shock protein 70, and transferrin (Fig. 2*H*), suggesting their involvement in host defense against PEDV or SIV infections. Based on these findings, we propose that the differential regulation of mucus secretion by nasal goblet cells may be a critical factor contributing to the distinct mucosal infection profiles of PEDV and SIV.

### PEDV and SIV Intestinal Mucosal Tropism Is Also Influenced by the Mucus Secretion Function of Intestinal Goblet Cells.

**Fig. 3.**
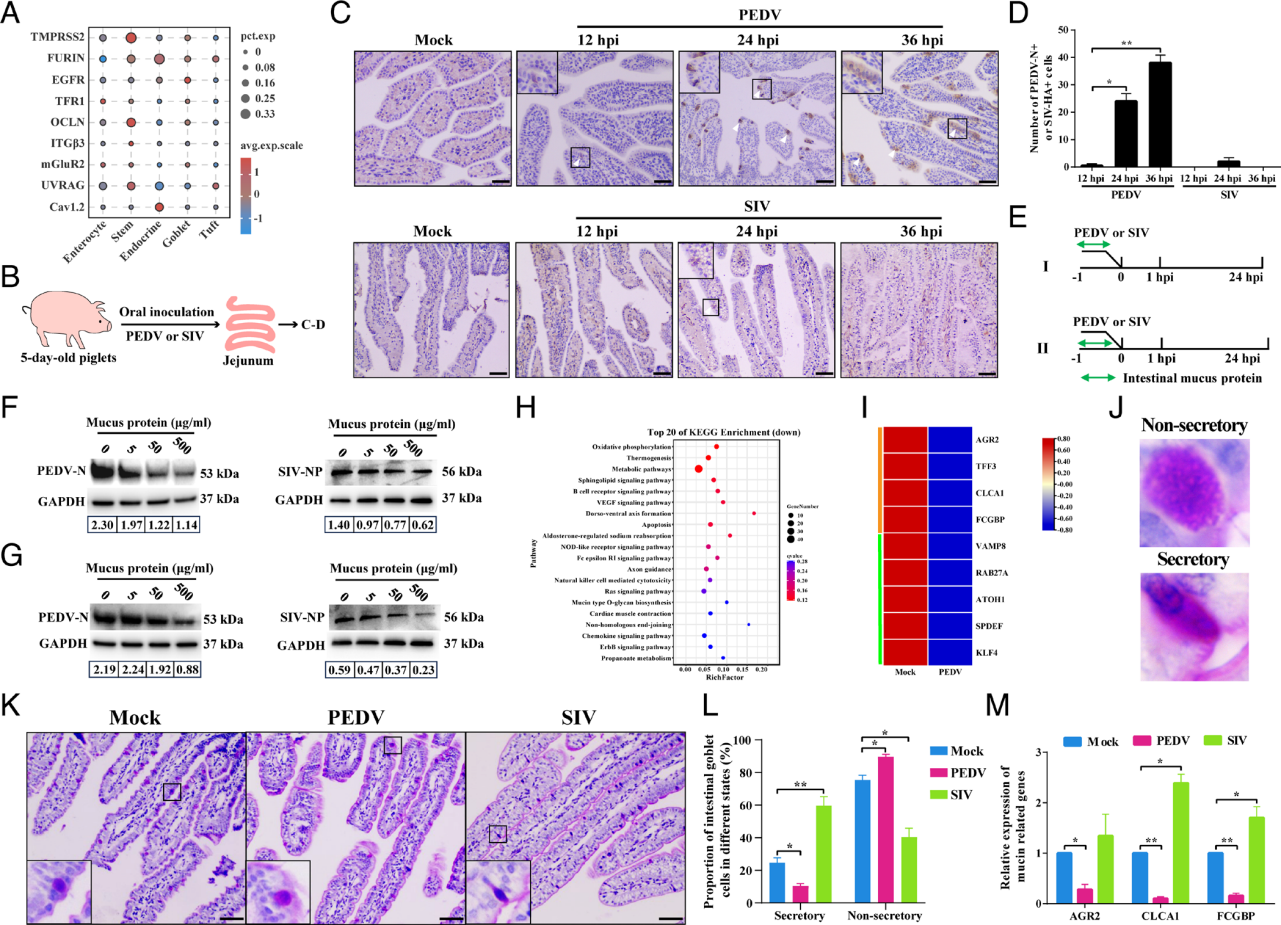
PEDV and SIV intestinal mucosal tropism is influenced by the mucus secretion function of small intestinal goblet cells. (*A*) Transcriptional profiles of host proteases and receptors involved in PEDV and SIV entry and infection across intestinal epithelial cell subsets. (*B*) Oral inoculation model used to assess PEDV and SIV replication in the jejunal mucosa of piglets. (*C*) IHC staining of PEDV and SIV distribution in the jejunum at 12, 24, and 36 hpi; (Scale bar, 20 μm.) (*D*) Quantification of PEDV^+^ or SIV^+^ epithelial cells in five randomly selected villi at different time points. (*E*) Experimental design to determine the infection stage at which total jejunal mucus protein inhibits PEDV or SIV: I, virus pretreatment; II, cell pretreatment. (*F* and *G*) Western blot analysis of antiviral activity of jejunal mucus protein after virus pretreatment (*F*) or cell pretreatment (*G*); numbers indicate the grayscale ratio of viral to GAPDH. (*H*) KEGG enrichment analysis of downregulated genes in goblet cells after PEDV infection. (*I*) Transcriptional features of mucus-associated proteins (orange module) and mucus secretion-related genes (green module) in goblet cells. (*J*) Morphology of nonsecretory and secretory goblet cells in the jejunum. (*K*) Representative PAS-stained jejunal sections from different treatment groups; (Scale bar, 20 μm.) (*L*) Proportions of secretory and nonsecretory goblet cells among total goblet cells in panel *J* (four random fields per section). (*M*) Relative expression of mucin-related genes in the jejunal mucosa from different groups. Data are mean ± SD from three independent experiments; *P < 0.05, **P < 0.01.

Consistent with observations in NECs, host proteases (TMPRSS2, FURIN) and virus entry-related receptors or cofactors associated with PEDV or SIV infection (e.g., EGFR, TFR1, OCLN, ITGβ3, mGluR2, UVRAG, and Cav1.2) were also broadly expressed at the transcriptional level across all epithelial subpopulations in the small intestine (Fig. 3*A*). Despite this, the two viruses showed distinct intestinal infection dynamics. PEDV spread progressively, from sparse positive cells at 12 hpi to widespread infection by 36 hpi, breaching the epithelial barrier into the lamina propria. SIV produced only transient, low-level infection, with limited signals between 12 to 24 hpi that declined sharply by 36 hpi (Fig. 3 *B*–*D*).

Given our earlier hypothesis that differences in viral tropism are closely tied to goblet cell function in the nasal mucosa, we investigated whether intestinal goblet cell mucus secretion underlies the divergent outcomes of PEDV or SIV infections. We observed that the total intestinal mucus proteins significantly inhibited PEDV or SIV infections, whether applied directly to the virus or preincubated with host cells (Fig. 3 *E*–*G*). scRNA-seq showed that PEDV markedly suppressed mucin synthesis and secretion pathways in intestinal goblet cells (Fig. 3*H*), downregulating the transcription of mucin-associated protein genes, including AGR2, CLCA1, and FCGBP (Fig. 3*I*). To assess mucus secretion changes, PAS and immunofluorescence staining classified goblet cells as secretory or nonsecretory based on mucin or Muc2 accumulation/release (Fig. 3*J* and *SI Appendix*, Fig.S4*C*). In controls, secretory cells comprised ~25% of goblet cells; PEDV reduced this to ~13%, while SIV increased it to ~32% (Fig. 3 *K* and *L* and *SI Appendix*, S4 *C* and *D*). Consistently, RT-qPCR confirmed PEDV downregulated and SIV upregulated AGR2, CLCA1, and FCGBP (Fig. 3*M*). Thus, SIV enhances, whereas PEDV suppresses, intestinal goblet cell mucus secretion—differences likely contributing to their distinct intestinal infection profiles.

### PEDV Induces the Formation of Goblet Cell-Associated Antigen Passages (GAPs) in Small Intestinal Goblet Cells.

scRNA-seq of PEDV-infected piglets revealed strong induction of genes linked to GAP formation (24), including those in cholinergic receptor muscarinic 3 (CHRM3), PI3K-AKT, actin polymerization, microtubule transport, and endocytosis pathways (*SI Appendix*, Fig. S5*A*). GAPs normally deliver soluble dietary antigens to promote immune tolerance and can be visualized in vivo with fluorescent dextran tracers (25).

**Fig. 4.**
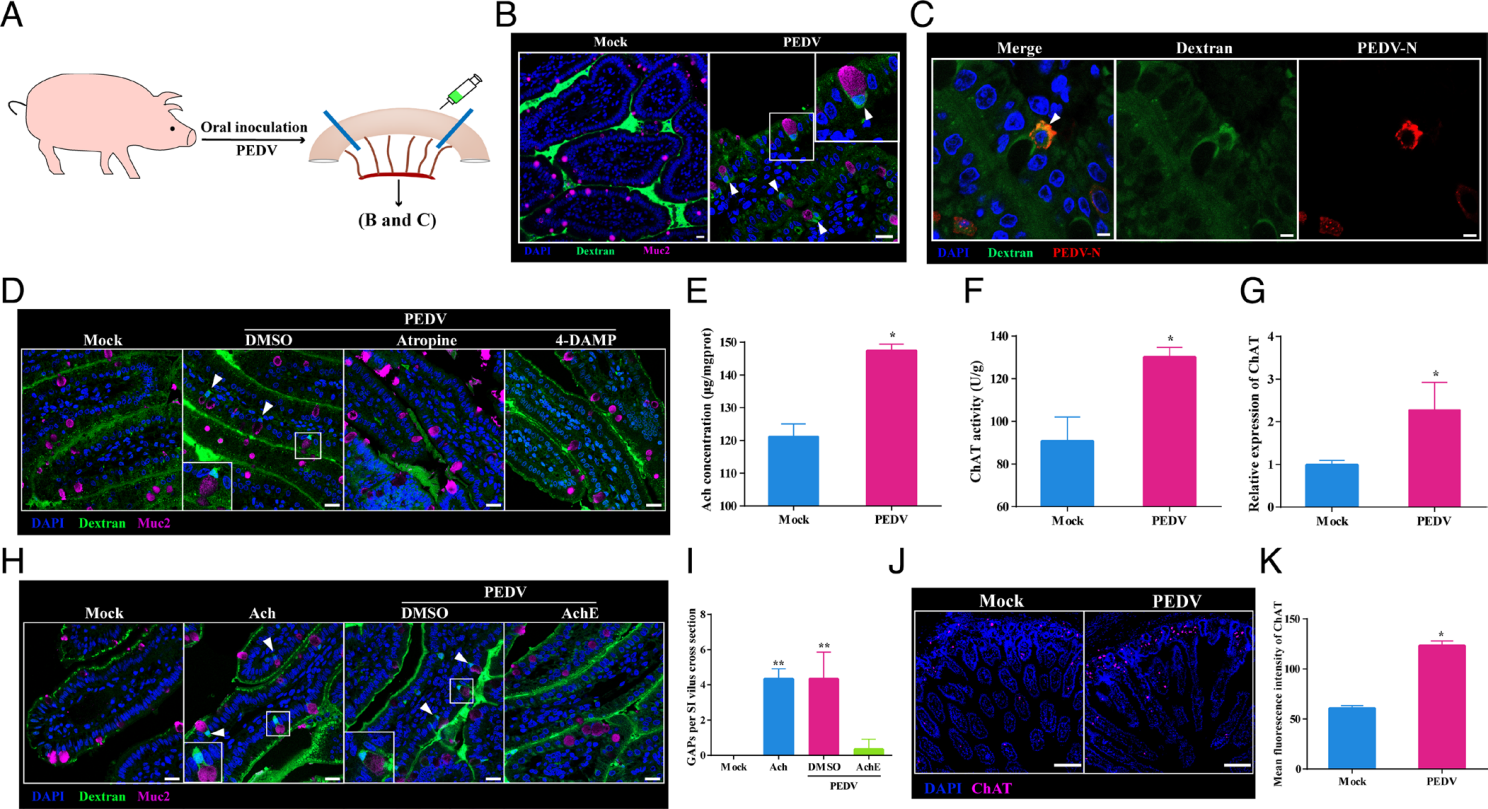
Interaction between ACh and CHRM3 drives the formation of GAPs. (*A*) Schematic of the piglet intestinal ligation model established to assess GAP formation in the small intestine after oral PEDV infection. (*B*) Confocal microscopy showing FITC-dextran (green) uptake into goblet cells (Muc2, pink) in the small intestine 48 h post-PEDV infection; nuclei stained with DAPI (blue). (Scale bar: 20 μm.) (*C*) Confocal images showing colocalization of PEDV (red) with GAPs (green) in the small intestine; nuclei stained with DAPI (blue). (Scale bar: 5 μm.) (*D*) Confocal microscopy showing GAP formation (FITC-dextran, green) in goblet cells (Muc2, pink) of the piglet small intestine under different treatment conditions (Mock, DMSO, Atropine, 4-DAMP). Nuclei are stained with DAPI (blue). (Scale bar: 20 μm.) (*E* and *F*) ELISA quantification of the effects of PEDV infection on ACh concentration (*E*) and choline acetyltransferase (ChAT) activity (*F*) in piglet intestinal mucosa. (*G*) RT-qPCR analysis of ChAT mRNA expression in the piglet small intestine after PEDV infection. (*H*) Confocal microscopy of GAP formation under different treatments (Mock, ACh, DMSO, AChE inhibitor); nuclei stained with DAPI (blue), goblet cells labeled with Muc2 (pink). (Scale bar: 20 μm.) (*I*) Quantification of GAP numbers per villus cross-section in panel *H* (three sections per group, 15 villi per section). (*J*) Fluorescent visualization of ChAT (pink) distribution in the piglet small intestine using a ChAT-specific probe; nuclei stained with DAPI (blue). (Scale bar: 50 μm.) (*K*) Quantification of mean ChAT fluorescence intensity from panel *J* (three sections per group, three fields per section, field area = 0.096 mm^2^). Data are shown as mean ± SD from three independent experiments. *P < 0.05, **P < 0.01.

To investigate whether PEDV induces GAPs in the small intestine, we established a piglet intestinal ligation model 48 h after oral infection and injected 10 kDa Fluorescein Isothiocyanate (FITC)-labeled dextran (Fig. 4*A*). Confocal and transmission electron microscopy revealed that FITC-dextran was localized in Muc2-positive goblet cells in PEDV-infected intestines (Fig. 4*B* and *SI Appendix*, Fig. S5 *B* and *C*), indicating GAP formation. Moreover, unlike mice, where soluble antigens transported via intestinal GAPs are partially degraded in lysosomes and partially trafficked to the Golgi apparatus for transcytotic release (24), we found that in piglet intestinal goblet cells, nearly all soluble antigens transported through GAPs were directed to the Golgi apparatus (*SI Appendix*, Fig. S5*D*). This suggests that GAPs in the piglet intestine possess a more efficient transcytotic transport capacity. Notably, PEDV particles also colocalized with FITC-dextran in GAP-positive goblet cells (Fig. 4*C*), implying direct PEDV transport to the lamina propria via GAPs. Further experiments showed that PEDV rapidly induced GAP formation as early as 2 hpi, whereas SIV did not. Neither inactivated PEDV nor the vaccine strains were capable of inducing GAP expression (*SI Appendix*, Fig. S5*E*).

### PEDV Drives the Formation of Small Intestinal GAPs through Acetylcholine (ACh)-CHRM3 Signaling.

Intestinal GAP formation is regulated by factors such as ACh, microbial sensing, and inflammatory mediators. In mice, dietary antigens induce GAPs via ACh-CHRM4 signaling (24). In the present study, although CHRM4 was not detected in porcine small intestinal goblet cells, we observed an 18-fold increase in CHRM3 transcription following PEDV infection (*SI Appendix*, Figs. S5*A* and S6*A*).

To investigate whether CHRM3 activation in porcine goblet cells was required for PEDV-induced GAP formation, we inhibited CHRM3 activity using specific antagonists. Both atropine, a broad-spectrum CHRM antagonist, and 4-Diphenylacetoxy-N-methylpiperidine methiodide (DAMP), a CHRM3-specific antagonist, significantly suppressed GAP formation in PEDV-infected piglets (Fig. 4*D*). To determine whether ACh contributed to PEDV-induced GAP formation, we measured ACh secretion in the small intestine after infection. PEDV infection significantly increased ACh secretion at 2 hpi (Fig. 4*E*). Meanwhile, the activity and transcription of choline acetyltransferase (ChAT), a key enzyme in ACh synthesis, was notably increased in PEDV-infected piglets (Fig. 4 *F* and *G*). Furthermore, exogenous ACh induced GAPs, while acetylcholinesterase treatment blocked PEDV-induced GAPs (Fig. 4 *H* and *I*), indicating that PEDV drives GAP formation by stimulating ACh release and activating CHRM3 in goblet cells. Fluorescence in situ hybridization (FISH) for ChAT transcripts was used to identify the primary source of ACh in the intestinal mucosa, revealing marked upregulation in PEDV-infected piglets, with ChAT-positive cells mainly in the submucosal layer (Fig. 4 *J* and *K*).

### PEDV Promotes the Release of ACh from Small Intestinal Nerve Cells, Thereby Inducing the Formation of Small Intestinal GAPs.

**Fig. 5.**
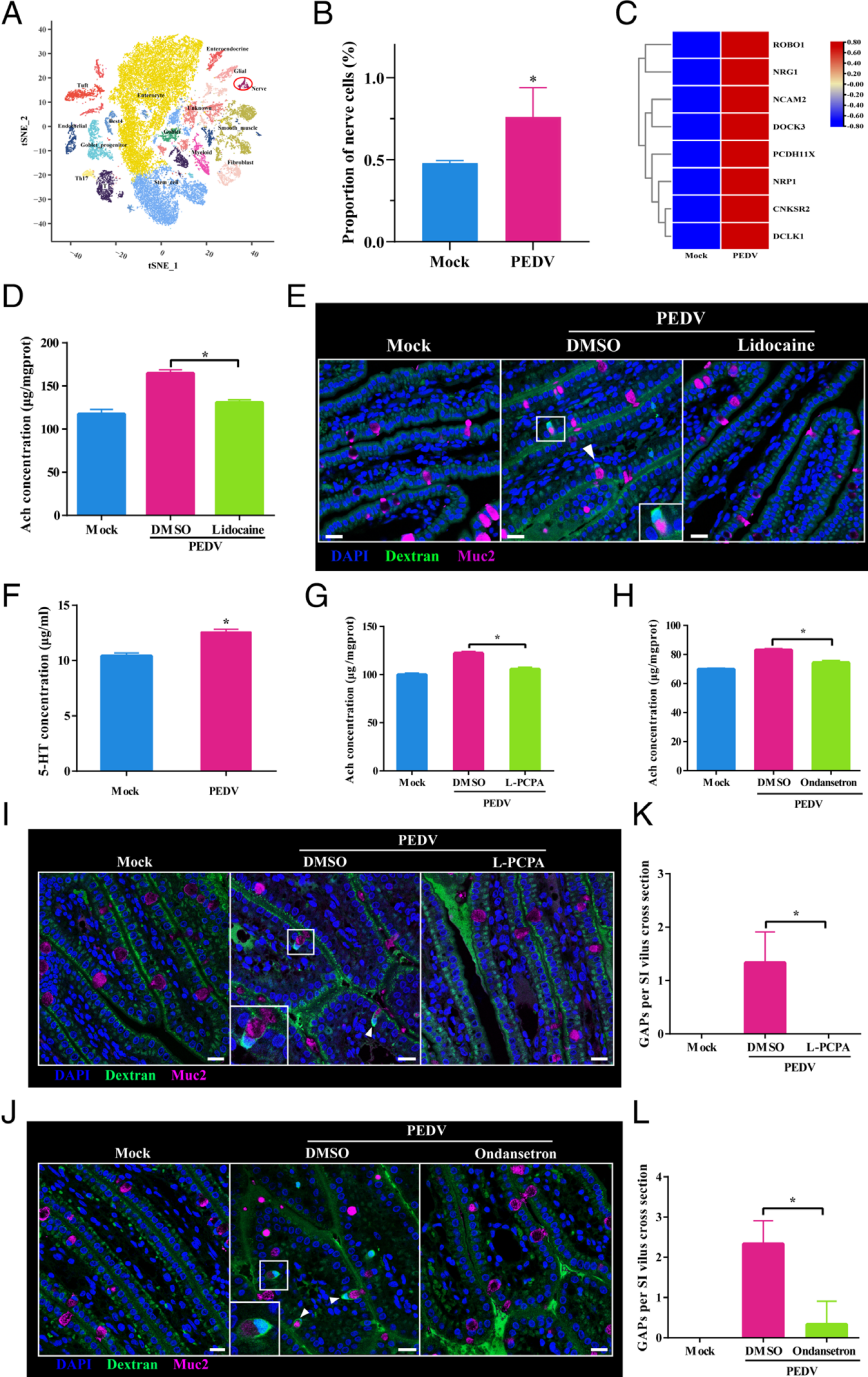
PEDV induces 5-HT release by endocrine cells, leading to neuronal ACh secretion and consequent GAPs formation. (*A*) t-SNE plot with annotated cell types from piglet small intestine tissue. (*B*) Proportion of intestinal neural cells in Mock versus PEDV-infected piglets. (*C*) Heatmap showing transcriptional profiles of neural activation–related genes in intestinal neural cells. (*D*) ELISA quantification of ACh concentration in small intestine tissue under different treatments. (*E*) Confocal microscopy of GAPs (FITC-dextran, green) in goblet cells (Muc2, pink) under various treatments; nuclei stained with DAPI (blue). (Scale bar: 50 μm.) (*F*) ELISA of 5-HT concentration in the intestinal mucosa following PEDV infection. (*G* and *H*) ELISA quantification of ACh levels in intestinal tissue under indicated treatments. (*I* and *J*) Confocal imaging of GAPs (FITC-dextran, green) in goblet cells (pink) under different treatment conditions; nuclei stained with DAPI (blue). (Scale bar: 20 μm.) (*K* and *L*) Quantitative analysis of GAP numbers per villus cross-section in (*I* and *J*), calculated from three sections with 15 villi per section. Data are shown as mean ± SD from three independent experiments. *P < 0.05.

In the intestine, ACh is mainly produced by T, tuft, and nerve cells, with nerve cells predominantly located in the submucosa (26, 27). To investigate whether ACh production following PEDV infection comes from nerve cells, we isolated total mucosal cells from piglet small intestines 2 hpi and performed single-nucleus RNA sequencing (snRNA-Seq). We used t-distributed stochastic neighbor embedding analysis to cluster these cells into 15 distinct subpopulations, including epithelial, goblet, and nerve cells (Fig. 5*A*). Notably, nerve cell abundance increased significantly after PEDV infection (Fig. 5*B*), accompanied by significant upregulation of neuron activation-associated genes (NRG1, NRP1, and DCLK1) (Fig. 5*C*). SnRNA-seq further revealed that porcine small intestinal nerve cells specifically expressed ELAVL3 and ENO2, which was confirmed by the synthesis of FISH probes targeting these genes. In PEDV-infected piglets, ChAT transcription was predominantly localized in submucosal nerve cells with high ELAVL3 and ENO2 expression (*SI Appendix*, Fig. S6 *B* and *C*).

As lidocaine, a local anesthetic, blocks sodium ion channels and thereby inhibits intestinal nerve signaling, we pretreated the piglet small intestines with 2% lidocaine before PEDV inoculation. Lidocaine pretreatment significantly reduced ACh levels postinfection (Fig. 5*D*) and suppressed PEDV-induced GAP formation (Fig. 5*E*). These results indicate that PEDV infection activates the submucosal nerve cells, promotes ACh secretion, and subsequently induces GAP formation in the piglet small intestine.

### PEDV-Induced Nerve Cell ACh Secretion Is Modulated by Endocrine 5-HT Release and Nav1.8 Nociceptor Activation.

Under physiological conditions, epithelial-derived neurotransmitters, such as 5-HT, are classic regulators of intestinal neuron activation (28). Here, we showed that PEDV infection triggered rapid 5-HT secretion in the small intestines of piglets as early as 2 hpi, paralleling the rapid formation of GAPs (Fig. 5*F*). To clarify the role of 5-HT in PEDV-induced GAP formation, piglets were pretreated with 4-chloro-l-phenylalanine (L-PCPA, a 5-HT synthesis inhibitor) and ondansetron (a 5-HT receptor antagonist). Both treatments significantly reduced ACh levels in the small intestinal mucosa (Fig. 5 *G* and *H*) and suppressed PEDV-induced GAP formation (Fig. 5 *I*–*L*).

Intestinal 5-Hydroxytryptamine (5-HT) is primarily synthesized and released by tuft and enteroendocrine cells, which serve as sentinel cells that respond to diverse stimuli by releasing neurotransmitters such as 5-HT (29, 30). This mechanism is essential for the regulation of neuronal activation and maintenance of intestinal homeostasis. Single-cell sequencing data from PEDV-infected piglets showed that tuft and enteroendocrine cells upregulated metabolic and biosynthetic pathways (*SI Appendix*, Fig. S6 *D*–*G*). However, only enteroendocrine cells exhibited significant Mitogen-Activated Protein Kinase (MAPK) and estrogen pathway activation, which is strongly associated with 5-HT synthesis. Moreover, these cells showed significantly increased expression of the key 5-HT synthesis genes TPH1 and DDC (*SI Appendix*, Fig. S6*H*). Single-cell transcriptomics revealed that porcine small intestinal enteroendocrine cells specifically express ChgA. Accordingly, we performed double staining for ChgA and 5-HT, which demonstrated that ChgA-positive enteroendocrine cells consistently expressed 5-HT in the small intestine (*SI Appendix*, Fig. S6*I*). Collectively, these findings indicate that enteroendocrine cell-derived 5-HT is critical for activating submucosal neurons in the PEDV-infected small intestine, thereby facilitating the formation of GAPs.

In addition to the “indirect activation” mechanism mediated by 5-HT, intestinal neurons directly sense the luminal environment through various receptors. In this study, we used the selective antagonists AMG517 and A803467 to block the nociceptors transient receptor potential cation channel subfamily V member 1 (TRPV1) and Nav1.8, respectively, to investigate their roles in the PEDV-induced formation of GAPs in the small intestine of piglets. The results showed that TRPV1 blockade had no significant effect on the number of intestinal GAPs in PEDV-infected piglets, whereas Nav1.8 inhibition significantly suppressed PEDV-induced GAP formation (*SI Appendix*, Fig. S6 *J* and *K*).

### Intestinal GAPs Are Essential in PEDV Induced Intestinal Mucosal Damage.

Under physiological conditions, GAPs induce immune tolerance to dietary antigens. However, when the mucus barrier is compromised or immature, pathogens, such as *Salmonella* and *Listeria*, can exploit GAPs to breach the epithelium, causing acute inflammation (31, 32). In neonates, breast milk–derived EGF activates EGFR in goblet cells to reduce cholinergic sensitivity and inhibit GAP formation (33). Here, we found that PEDV infection significantly suppressed EGFR phosphorylation in goblet cells within 2 hpi (*SI Appendix*, Fig. S7 *A* and *B*), indicating that PEDV induces GAP formation via submucosal neuron activation and disrupts key signaling pathways that repress GAPs.

**Fig. 6.**
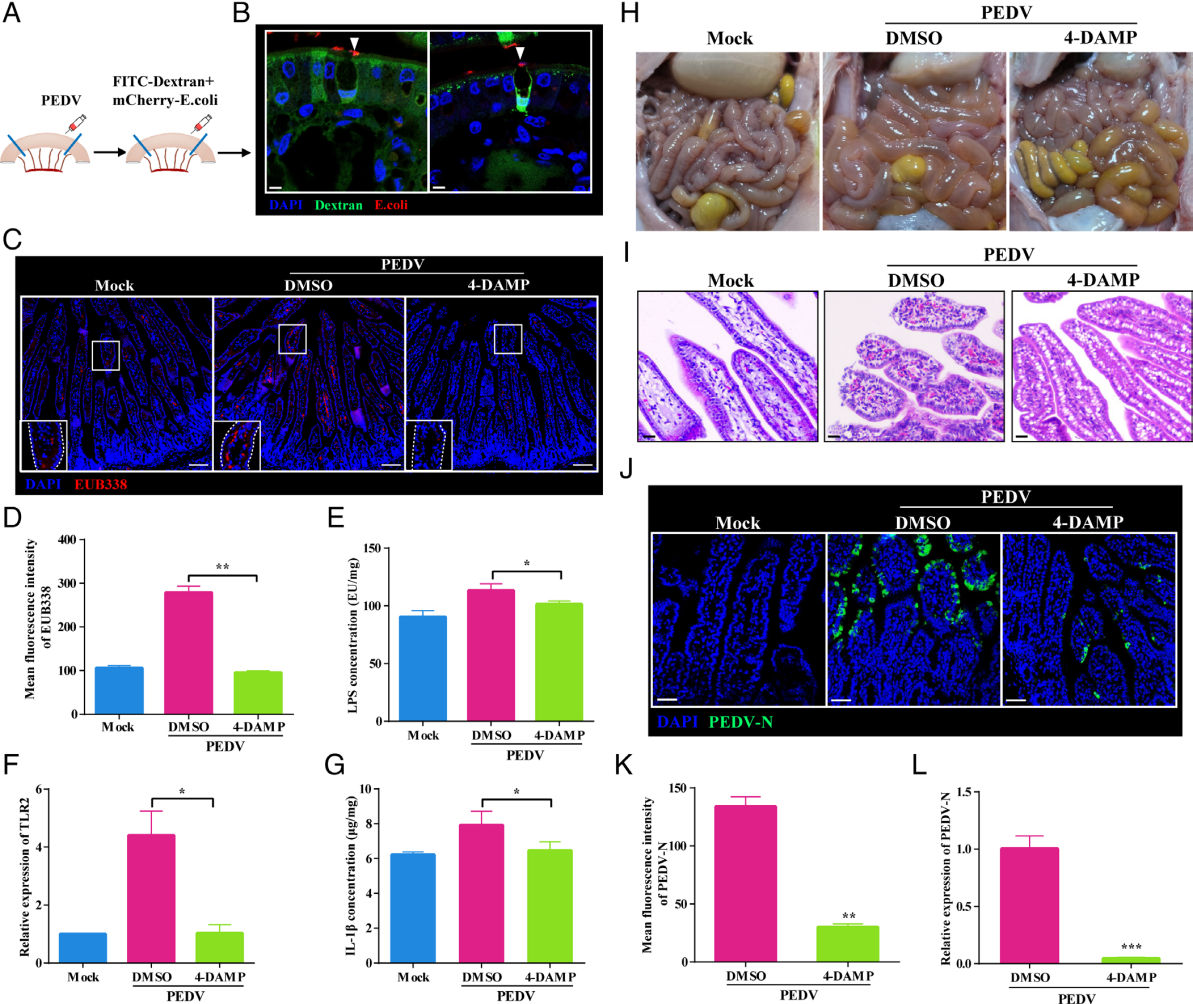
GAPs are crucial for the establishment of intestinal mucosal infections by PEDV. (*A*) Schematic of the intestinal ligation model used to assess the impact of GAPs on bacterial invasion in piglets. (*B*) Confocal microscopy showing uptake of FITC-dextran (green) and mCherry-*E. coli* (red) by goblet cells; nuclei stained with DAPI (blue). (Scale bar: 5 μm.) (*C*) Confocal images of intestinal bacteria detected using the universal bacterial probe EUB338 (red), showing bacterial penetration into the lamina propria; nuclei stained with DAPI (blue). (Scale bar: 50 μm.) (*D*) Quantification of mean fluorescence intensity of EUB338 in panel *C* (three sections, 15 villi per section). (*E*) ELISA measurement of LPS concentration in intestinal tissue across treatments. (*F*) RT-qPCR analysis of TLR2 expression in the small intestine. (*G*) ELISA measurement of IL-1β concentration in the small intestine. (*H*) Gross pathology of piglet intestines under different treatments. (*I*) Hematoxylin and Eosin staining of the small intestine under different treatments. (Scale bar: 20 μm.) (*J*) Confocal microscopy showing PEDV (green) distribution in the small intestine; nuclei stained with DAPI (blue). (Scale bar: 50 μm.) (*K*) Quantification of PEDV fluorescence intensity in panel *J*. (*L*) RT-qPCR analysis of PEDV gene expression in the small intestine. Data are mean ± SD from three independent experiments. *P < 0.05, **P < 0.01, ***P < 0.001.

To assess whether PEDV-induced GAPs facilitate bacterial translocation, we used a ligated intestinal loop model in piglets with mCherry-expressing *Escherichia coli* (Fig. 6 *A* and *B*). After PEDV infection, bacteria preferentially entered GAP-positive goblet cells. Despite intact epithelial tight junctions (*SI Appendix*, Fig. S7 *C*–*F*), bacteria infiltrated the lamina propria (Fig. 6 *C* and *D*), increasing lipopolysaccharide (LPS) levels (Fig. 6*E*). scRNA-seq and FISH showed that PEDV upregulated TLR2 (Fig. 6*F*), activating macrophage TNF and NF-κB pathways and elevating IL-1β, IL-18, and IL-6 (Fig. 6*G* and *SI Appendix*, Fig. S7 *G*–*J*). Blocking GAPs with 4-DAMP reduced bacterial load, LPS levels, IL-1β secretion, and TLR2 transcription in macrophages (Fig. 6 *C*–*G* and *SI Appendix*, Fig. S7 *I* and *J*). These findings indicate that PEDV rapidly induces GAPs in neonatal piglets, enabling bacterial translocation into the lamina propria without disrupting epithelial tight junctions, thereby initiating localized inflammation.

We conducted a challenge experiment to examine the effects of GAP inhibition on PEDV pathogenicity. Clinical observations showed that pharmacological inhibition of GAP formation with 4-DAMP markedly reduced PEDV-induced disease severity in piglets (*SI Appendix*, Fig. S8 *A* and *B* and Table S2). In the DMSO group, watery diarrhea (score 2 to 3) appeared at 1 dpi, peaked from 2 dpi onward, and was accompanied by severe appetite loss, lethargy, vomiting (≥2 episodes/d on 3 to 4 dpi), and progressive weight loss (~22% by 5 dpi). In contrast, 4-DAMP-treated piglets developed only mild, delayed diarrhea (score ≤2), preserved appetite and activity, minimal transient weight loss (~3% at 2 dpi), and recovered rapidly. Mock-inoculated piglets remained clinically normal throughout the study, exhibiting no signs of diarrhea, weight loss, or behavioral changes. Necropsy revealed intestinal wall thinning, luminal fluid accumulation, and severe villus atrophy with lamina propria erythrocyte infiltration in DMSO-treated piglets, whereas 4-DAMP-treated animals had milder lesions and largely intact villi (Fig. 6 *H* and *I*). Immunofluorescence and RT-qPCR confirmed markedly fewer PEDV-positive cells and lower viral loads in the intestines of 4-DAMP–treated piglets (Fig. 6 *J*–*L*). These results indicate that GAP inhibition mitigates PEDV-induced intestinal mucosal damage and alleviates clinical symptoms.

Extensive studies indicate that PEDV is more pathogenic in neonatal piglets than transmissible gastroenteritis virus (TGEV) or porcine deltacoronavirus (PDCoV), with mortality rates up to 100%. Using an oral infection model with identical viral doses and observation intervals, we observed earlier and more severe disease in PEDV-infected piglets (*SI Appendix*, Fig. S8 *C* and *D*). All three viruses caused severe watery diarrhea (score 3), but onset occurred at 1 dpi only in PEDV, versus 2 dpi in TGEV and PDCoV. Weight loss was greatest in PEDV-infected animals (*SI Appendix*, Fig. S8*D*). Histopathology showed more extensive villus atrophy and shedding in PEDV-infected intestines, while TGEV and PDCoV produced only mild lesions (*SI Appendix*, Fig. S8 *E* and *F*). Intestinal ligation experiments confirmed that neither TGEV nor PDCoV induced GAP formation (*SI Appendix*, Fig. S8*G*), suggesting that PEDV’s ability to trigger GAPs may contribute to its heightened pathogenicity in neonatal piglets.

## Discussion

Current studies on viral mucosal infections focus on “susceptible cells” that support viral entry and replication, while overlooking the roles of nonsusceptible cells—such as epithelial, lamina propria, and immune cells—in regulating colonization and pathogenesis. In this study, we compared nasal mucosal infection by a respiratory virus (SIV) and an enteric virus (PEDV). Both infected NECs, yet PEDV caused brief, localized infection, whereas SIV replicated efficiently and spread. scRNA-seq showed that ciliated cells—the primary initial targets—expressed entry receptors and proteases for both viruses, indicating that differences in infection outcomes are not solely determined by susceptible cells.

Although SIV and PEDV did not markedly change the overall immune cell composition in the nasal mucosa, they triggered distinct mucosal immune responses. SIV induced a strong innate response, likely because its sustained replication releases large amounts of PAMPs, driving DAMP and cytokine production and amplifying local signaling (34). In contrast, PEDV’s limited replication fails to cause extensive tissue damage or inflammation. SIV also employs immune evasion strategies—such as downregulating Interferon α Receptor, inhibiting Janus Kinase–Signal Transducer and Activator of Transcription (JAK-STAT) signaling, and disrupting Interferon-stimulated growth factor 3 formation—to avoid antiviral responses (35), explaining how it can elicit strong immune activation while maintaining colonization. For PEDV, the innate response was largely mediated by epithelial cells, with minimal lamina propria activation, suggesting infection remains restricted to the epithelial compartment. Overall, these results highlight the central role of NECs in shaping mucosal immunity to SIV and PEDV and provide clues to the mechanisms underlying their different nasal infection patterns.

Cellular communication analysis showed that PEDV infection enhanced interactions among cycling basal, ciliated, and goblet cells and with other epithelial types, whereas SIV suppressed them. Such crosstalk is essential for maintaining mucosal physical and chemical barriers: Basal cells mediate repair, ciliated cells clear pathogens, and goblet cells secrete protective mucus (23). Although SIV persists in nasal mucosa, its effect on basal and ciliated cells is weaker than PEDV’s, consistent with SIV’s tendency to colonize the upper respiratory tract before spreading to the lungs (14). PEDV downregulated genes for cell proliferation and tight junctions, indicating epithelial barrier compromise despite inefficient infection, and upregulated genes for ciliary formation and motility. While increased ciliary activity generally promotes clearance, some viruses can exploit cilia for infection (36), suggesting that PEDV-induced transcriptional reprogramming of ciliated cells warrants further study.

Transcriptional changes in nasal goblet cells are closely linked to the mucosal infection patterns of SIV and PEDV. In vivo, PEDV enhanced mucus secretion, aiding early viral clearance, whereas SIV suppressed it, promoting dissemination. Although nasal mucus is a key respiratory defense, its antiviral function is not fully defined. We found that porcine nasal mucus contains complement C3, galectin, serpin, transferrin, and calpain-1, which neutralize both viruses by blocking receptor binding, reducing progeny infectivity, and recruiting immune cells (37, 38). Given that mucus secreted by goblet cells in the porcine intestine also exhibits potent antiviral activity (39), we examined the gut and observed similar patterns: PEDV suppressed mucus to sustain replication, while SIV induced it to limit infection. Thus, goblet cell function critically shapes PEDV and SIV outcomes at different mucosal sites. Goblet cells detect pathogens through pattern recognition receptors (Toll-Like Receptors, NOD-Like Receptors, RIG-I), which induce mucus protein expression and pathogen clearance (40, 41). PEDV infection of the nasal mucosa did not activate these receptors but instead induced IL-17 and IL-22 expression in immune cells, promoting mucus synthesis via JAK-STAT3 and MAPK pathways (42, 43), suggesting indirect regulation. In contrast, SIV suppressed NF-κB, PI3K-AKT, and ROS pathways which involved in mucus protein transcription and secretion in nasal goblet cells (44, 45); single-cell data suggest it can enter these cells, though direct manipulation remains unproven. Notably, our findings reveal that PEDV markedly suppresses intestinal goblet cell secretion through the inhibition of NOD-like receptor signaling and mucin protein synthesis pathways. Previous studies employing neonatal piglet infection models and porcine intestinal enteroids demonstrated that goblet cells are susceptible to PEDV infection (46, 47). Therefore, PEDV may modulate these pathways upon goblet cell infection, though the exact mechanisms remain unclear. We also observed strong activation of apoptosis- and necroptosis-related pathways in goblet cells after oral PEDV challenge, explaining the rapid depletion of these cells in villus and crypt regions during early infection (47). Collectively, these findings highlight the multiple strategies employed by PEDV—including suppression of mucus secretion and induction of goblet cell death—to compromise the intestinal mucus barrier and exacerbate pathogenicity in neonatal piglets.

In addition to suppressing intestinal goblet cell mucus secretion, PEDV also activates goblet cell signaling pathways that promote GAP formation. Under normal conditions, GAPs deliver dietary and commensal antigens to CD103^+^ dendritic cells in the lamina propria, inducing Foxp3^+^ Treg differentiation and maintaining intestinal immune tolerance (25). Although pathogens such as *Salmonella* and *Listeria* can exploit GAP-mediated transcytosis to invade the mucosa (31, 32), goblet cells normally sense microbes through a MyD88-dependent pathway and inhibit GAP formation through EGFR signaling, especially in neonatal intestines (33). We observed no GAPs in healthy neonatal pig intestines, but PEDV rapidly induced them early in infection, likely by suppressing EGFR phosphorylation in goblet cells. At 12 h postoral PEDV infection, the epithelial barrier remained intact, yet bacteria translocated via PEDV-induced GAPs into the lamina propria, triggering macrophage-mediated inflammation. Blocking GAP formation significantly reduced bacterial load, inflammation, and PEDV replication. The severity and clinical outcomes of viral infections are often substantially impacted by secondary bacterial infections, which can contribute more to mortality than the virus itself. A well-known example is the 1918 Spanish influenza pandemic, during which most fatalities were due to secondary bacterial pneumonia caused by *Streptococcus pneumoniae* or *Staphylococcus aureus* (48). Similarly, among elderly hospitalized or Intensive Care Unit (ICU)-admitted patients with RSV infection, secondary bacterial infections increase mortality risk approximately two- to threefold (49). Furthermore, infantile diarrhea induced by rotavirus or other enteric viruses often becomes severe due to secondary infection with *Salmonella* or pathogenic *E. coli*, resulting in enterocolitis, sepsis, and significantly increased mortality (50, 51). Notably, although the incidence of bacterial coinfections in SARS-CoV-2 is relatively low, multiple cohort studies show markedly higher hospital and ICU mortality when superinfections occur (52). Based on these findings, we hypothesize that PEDV infection in neonatal pig intestines is not merely a “local replication–spread” process. By inducing early GAP formation, PEDV enables gut bacteria to translocate into the lamina propria, triggering inflammation and barrier disruption that promote further viral infection and dissemination. Conversely, SIV and other porcine enteric coronaviruses, such as TGEV and PDCoV, do not induce GAP formation. Clinical and experimental evidence consistently shows that PEDV has markedly higher intestinal pathogenicity in neonatal piglets than these viruses, with mortality rates up to 100% (53), suggesting that GAP induction is a key mechanism underlying its virulence. Although TGEV does not induce GAPs, it can bind mucin-type glycoproteins on goblet cells via the sialic acid-binding activity of its spike protein, preventing clearance by peristalsis and enabling penetration of the mucus barrier to infect epithelial cells (54). These findings highlight the diverse strategies by which enteric viruses exploit goblet cells for mucosal infection and reinforce their pivotal role in intestinal pathogenesis.

Unlike mouse small-intestinal goblet cells, which require CHRM4 activation for GAP formation (24), piglet jejunal goblet cells show no CHRM4 transcription. Instead, PEDV induces GAP formation via CHRM3 activation, revealing species-specific differences in goblet cell cholinergic signaling. CHRM3, a G protein–coupled receptor, supports epithelial homeostasis and barrier integrity by promoting intestinal stem cell proliferation and differentiation while suppressing inflammation (55). Our results indicate that PEDV activates submucosal neurons to release ACh, which in turn stimulates CHRM3 in goblet cells. “Sentinel” cells such as enteroendocrine and tuft cells release serotonin (5-HT) and γ-aminobutyric acid early in enteric pathogen infection, helping maintain mucosal barrier homeostasis via the enteric nervous system (28, 56). In PEDV infection, early 5-HT release from enteroendocrine cells triggers neuronal ACh secretion, with upregulated pathways for 5-HT synthesis and release (e.g., MAPK, estrogen receptor, metabolism) suggesting PEDV-mediated modulation of 5-HT production. Because excess 5-HT can disrupt intestinal motility (57), this mechanism may contribute to vomiting and abdominal pain in infected piglets. In addition to the enteroendocrine and tuft cells, nerve endings in the submucosa detect various physical and chemical stimuli via nociceptors, thereby activating neuroimmune pathways that promote local anti-infection responses and tissue repair (58). We found that blocking Nav1.8, a sodium ion channel critical for amplifying nociceptive signals, significantly inhibited PEDV-induced GAP formation, suggesting that nociceptors are essential for the detection of early PEDV infection. Although TRPV1, a prototypical nociceptive receptor, has been reported to sense danger signals from various viruses (59), its inhibition did not affect PEDV-induced GAP formation, suggesting the involvement of other nociceptors in recognizing PEDV.

**Fig. 7.**
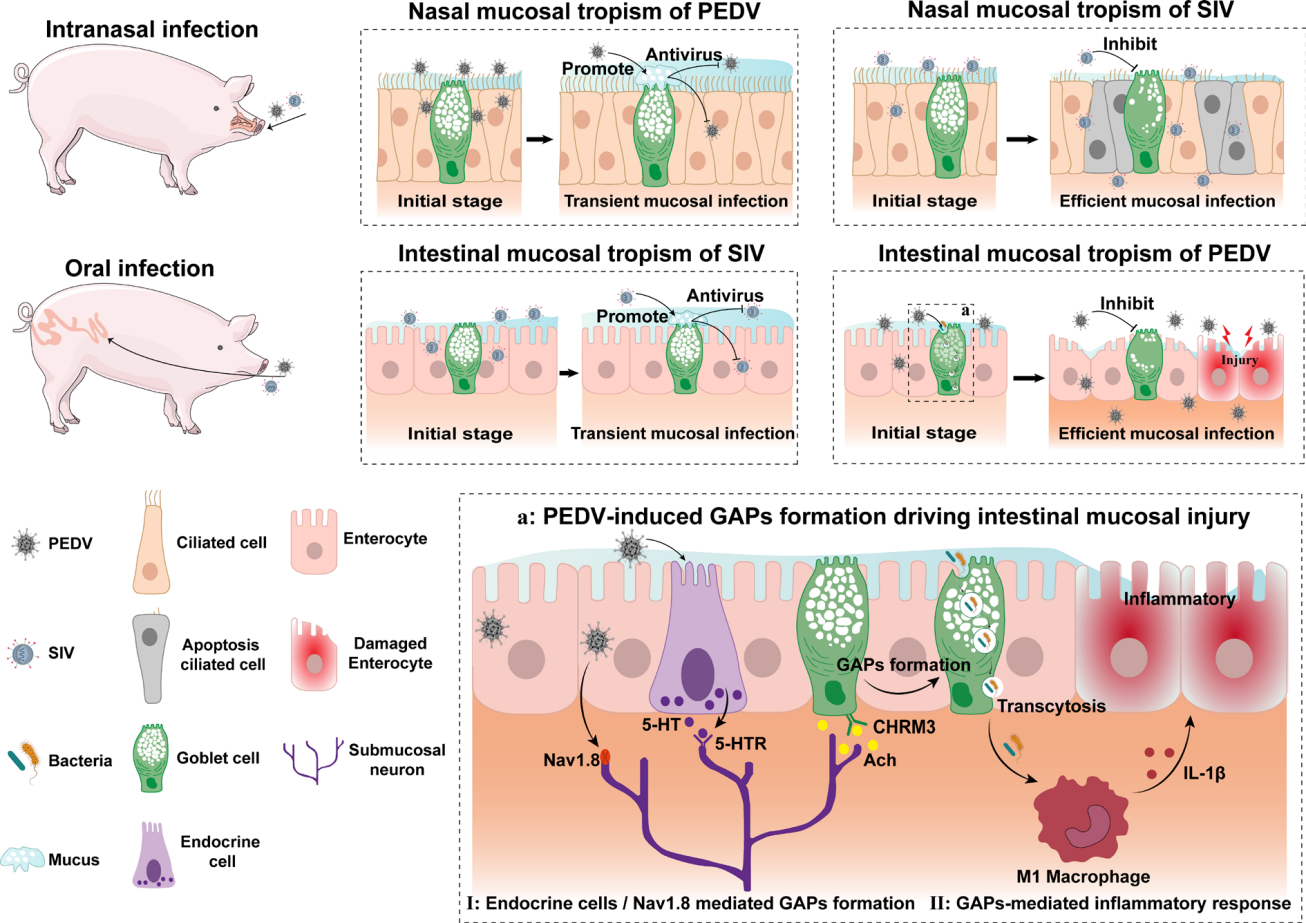
Schematic of goblet cell–mediated mucosal tropism and pathogenesis of SIV and PEDV.

By comparing the mucosal infection processes of SIV and PEDV, this study systematically elucidated the crucial regulatory role of nonsusceptible goblet cells in viral infection and dissemination at mucosal sites. This confirms that goblet cell mucus secretion influences infection outcomes and reveals that PEDV activates submucosal enteric neurons and induces GAP formation via CHRM3 signaling at an early stage of infection, translocating gut bacteria to the lamina propria, triggering inflammatory damage, and enhancing viral infection (Fig. 7). These findings advance our understanding of the multicellular interaction networks underlying SIV or PEDV infections at different mucosal sites and provide a theoretical basis for developing mucosal antiviral strategies targeting goblet cell function. Considering that numerous human pathogens, such as SARS-CoV-2 and rotavirus, establish low-level infections at multiple mucosal sites while showing clear mucosal tropism, further elucidation of the relationship between goblet cell function and mucosal infection outcomes could lead to the development of broad-spectrum antiviral strategies.

## Materials and Methods

A detailed description of the materials and methods used in the study is provided in *SI Appendix, Materials and Methods*. Reagents and antibodies, viruses, cell lines, bacterial strains, and animals were used in this study. Experimental procedures included virus challenge experiments, cell isolation, and single-cell library preparation. Downstream analyses comprised scRNA-seq data processing and quality control, differential expression analysis, pathway enrichment, and cell–cell communication analysis. Additional approaches involved single-nucleus RNA sequencing, assessment of viral effects on GAP formation and GAP-mediated bacterial translocation in piglet intestines, and investigation of GAP activation or inhibition in the piglet small intestine. Histological analysis, mucus preparation, total protein extraction, and LC–MS/MS were performed to characterize tissue and protein features. Functional assays included determination of the antiviral activity of mucus proteins, RT-qPCR, IHC, indirect immunofluorescence assay (IFA), fluorescence in situ hybridization (FISH), and ELISA.

## Supplementary Material

Appendix 01 (PDF)

## Data Availability

scRNA-seq data of the nasal mucosa from healthy piglets, as well as those infected with PEDV or SIV, are available under SRA accession number PRJNA1247933 (60). snRNA-seq data of the jejunum from piglets are available under SRA accession number PRJNA1248390 (61). Previously published data were used for this work (GSE175411) (62). All data supporting the findings of this work are available within the article and *SI Appendix*.
