# Supplementary material for: Goblet cells dictate viral tropism and pathogenesis in nasal and intestinal mucosae
Source: Proc Natl Acad Sci U S A. 2025 Oct 8;122(41):e2514150122. doi: 10.1073/pnas.2514150122 (PMC12541430; doi:10.1073/pnas.2514150122)
Supplement: Supplementary file 1 — Appendix 01 (PDF) [file pnas.2514150122.sapp.pdf]

Supplementary Information

Goblet cells dictate viral tropism and pathogenesis in nasal and intestinal mucosae

SI Appendix

Materials and Methods

Reagents and antibodies

4-DAMP (4-Diphenylacetoxy-*N*-methyl-piperidine methiodide), atropine, acetylcholine chloride, and acetylcholinesterase were purchased from Macklin Biochemical Technology (Shanghai, China). AMG517, A-803467, lidocaine, 4-chloro-L-phenalanine (L-PCPA), ondansetron, and FITC-dextran (MW 10000) were purchased from MedChemExpress LLC (NJ, USA). Diamidino-2-phenylindole (DAPI 1:1000, 2313070) was purchased from Thermo Fisher Scientific (Waltham, MA, USA). The anti-PEDV N protein monoclonal antibody was custom-made by Jinsirui Biotechnology Co., Ltd. (Nanjing, China) and stored in our laboratory (diluted 1:200 for immunofluorescence and 1:1000 for western blot analysis). The influenza A H1N1 nuclear protein/NP antibody was kindly provided by Professor Jihui Ping at the College of Veterinary Medicine, Nanjing Agricultural University, China. Mucin-2 (MUC2) antibody (abx177613) was purchased from Abcam (Cambridge, UK). Mucin 5AC (MUC5AC) antibody was purchased from Abcepta (Suzhou, China). The chromogranin A (CHGA) mouse monoclonal antibody (MBS439534) was obtained from MyBioSource (San Diego, CA, United States). The anti-serotonin antibody (ab6336) was purchased from Abcam (Cambridge, UK). Anti-LAMP1 antibody (bs-1970R) was purchased from Bioss (Beijing, China). The RAB7A polyclonal (55469-1-AP) and TGN46 monoclonal (66477-1-Ig) antibodies were purchased from Proteintech (Wuhan, China). The phospho-EGF receptor monoclonal antibody (3777T) was purchased from Cell Signaling Technology (Danvers, MA, USA). Occludin Polyclonal antibodies (GB111401) were obtained

from Servicebio (Wuhan, China). Secondary antibodies for immunofluorescence, including goat anti-mouse Alexa Fluor 488, goat anti-mouse Alexa Fluor 594, goat anti-rabbit Alexa Fluor 594, goat anti-rabbit Alexa Fluor 594, and a horseradish peroxidase-conjugated GAPDH monoclonal antibody (30203ES50), were purchased from Yeasen (Shanghai, China).

Viruses, cell lines, and bacterial strains

Vero E6, swine testes (ST), IPEC-J2, and MDCK cells were maintained in our laboratory. Cells were regularly tested for mycoplasma contamination and were cultured in Dulbecco's Modified Eagle's medium (DMEM) with 10% fetal bovine serum (FBS), and incubated at 37°C in a humidified atmosphere with 5% CO_2. The NECs culture protocol has been previously described(1). In brief, the acquired NECs were collected and seeded at a concentration of 2×10^5 cells cm^{-2} onto Transwell tissue culture inserts (3-μm pore size; 6.5-mm membrane diameter; Corning) coated with collagen from human placenta Type IV (6 μg mL^{-1}) (Sigma). After the cells reached confluence, the medium was replaced with a differentiating medium, and the cells were brought to the air–liquid interface. When NECs formed an air–liquid interface and has stable transepithelial electrical resistance, they were used for virus inoculation.

A highly virulent PEDV strain (GenBank accession no. KJ020932) was kindly provided by the Guangdong Haid Institute of Animal Husbandry and Veterinary Medicine. The PEDV vaccine strain was provided by the Veterinary Medicine Research Center of the Da Bei Nong Group. H1N1 (A/swine/Guangdong/1/2011, GenBank: MT410579) is a classical swine influenza virus kindly provided by Zhixin Feng (Jiangsu Academy of Agricultural Sciences). TGEV SHXB (GenBank: KP202848) was provided by the Jiangsu Academy of Agricultural Sciences. PDCoV CH/JX/JGS/01 (GenBank: KY293677) was kindly provided by the JiangXi Agricultural University. Heat-inactivated PEDV was prepared at 56°C for 30 min and tested for complete loss of infectivity by inoculation into Vero cells at a multiplicity of infection (MOI) = 1 for CPE observations. For viral inoculation, a

confluent monolayer of target cells was inoculated with virus at a MOI of 1 for 1 h at 37°C. The inoculum and unattached viruses were removed by washing the cells with DMEM. Maintenance medium (DMEM with 1% FBS) was then added, and the culture was incubated at 37°C under 5% CO_2. Infected cells were analyzed after the required incubation period. To harvest viruses, infected cells were subjected to one freeze-thaw cycle, achieving a cytopathic effect of 80%, and cell supernatant collected. All viral particles were titrated onto target cells using a plaque assay and preserved at −80°C. After growing into a confluent monolayer, NECs were infected with PEDV or SIV (MOI = 1) for 1 h at 37°C and washed extensively to remove unbound virus. After 12 and 24 h, the cells were collected for viral RNA titer determination. The mCherry-*E. coli* strain was kindly provided by Professor Zhe Ma at the College of Veterinary Medicine, Nanjing Agricultural University, China. The mCherry-*E. coli* strain was grown in Luria–Bertani (LB) media or on LB plates fortified with 1.5% agar at 37 °C and purified and counted before use.

Animals

Five-day-old neonatal piglets were used for PEDV challenge and ligated-loop experiments. The piglets in this study were sourced from a swine herd maintained by Peiqi Agricultural and Animal Husbandry Technology Co., Ltd. (Jiangsu, China), which is composed of cesarean-derived, colostrum-deprived piglets that test seronegative for antibodies against PEDV, PRRSV, TGEV, PDCoV, influenza A virus, and porcine respiratory coronavirus. To eliminate the potential influence of maternally derived antibodies, neonatal piglets were artificially fed throughout the experiment.

All animals used in the study were of comparable weight and housed individually in separate rooms for 24 h prior to the study to allow for acclimatization to husbandry conditions and to minimize stress. All animal experiments were approved by the Institutional Animal Care and Use Committee of the Nanjing Agricultural University Animal Experiment Ethics Committee (NJAULLSC2022060 and NJAULLSC2023151, Nanjing, China).

Virus challenge experiments

To investigate the mucosal infection characteristics of PEDV and SIV, intranasal and oral experiments were performed. For intranasal inoculation, piglets (5-day-old) of similar weights were randomly allocated to five groups (four piglets per group). The 5 groups were (I) Mock, (II) PEDV intranasal inoculation 12 h, (III) PEDV intranasal inoculation 24 h, (IV) SIV intranasal inoculation 12 h, and (V) SIV intranasal inoculation 24 h. Among them, piglet mucosae from the nasal respiratory region, with two porcine nasal mucosae mixed into one sample, from groups I, III, and V were used for single cell RNA sequencing. For oral inoculation, piglets (5-day-old) of similar weight were randomly allocated to seven groups (four piglets per group). The 7 groups were (I) Mock, (II) PEDV oral inoculation 12 h, (III) PEDV oral inoculation 24 h, (IV) PEDV oral inoculation 36 h, (V) SIV oral inoculation 12 h, and (VI) SIV oral inoculation 24 h. (VII) SIV oral inoculation 36 h. The intranasal inoculation doses of PEDV and SIV were 1 mL (10^6 PFU mL^{-1}), and the oral inoculation doses of PEDV and SIV were 1 ml (10^5 PFU ml^{-1}). The nasal and intestinal tissues were collected for subsequent experiments after the piglets were euthanized.

To compare the mucosal infection characteristics of PEDV, PDCoV, and TGEV, twenty-four newborn piglets of similar weights were randomly allocated to four groups and housed in four separate rooms. Except for the Mock group, piglets from other groups were challenged with 1 mL of PEDV, PDCoV, or TGEV (10^6 PFU/mL) by oral inoculation. Body weights were measured daily post-viral challenge, with fecal swab samples collected and fecal consistency scored. At 72 hpi, after all groups of piglets showed symptoms of diarrhea, half of the piglets were euthanized. At 5 dpi, the remaining piglets were euthanized, and necropsies were performed. The jejunum was removed, embedded in paraffin, and cut into 4-μm sections for pathological observation.

Cell isolation and single-cell library preparation

To isolate nasal mucosal cells, fresh nasal tissues were dissected into small pieces and digested in Type IV collagenase (4 mg/mL, Miltenyi Biotec) and hyaluronidase (0.25 mg/mL, Miltenyi Biotec) for 30 min at 37 °C. Following digestion, the released cells were filtered through a 70-μm cell strainer, centrifuged, and resuspended in PBS. Cell viability was assessed by trypan blue staining, and the live-cell density was adjusted to approximately 1,000–2,000 cells/μL. Single-cell capture was performed using a Chromium Controller (10 × Genomics) following the manufacturer's instructions. The barcoded gel beads were bound to the cells, and reverse transcription and library construction were completed. Raw scRNA-seq data were generated by Gene Denovo (Guangzhou, China).

scRNA-seq data processing and quality control

Raw sequencing reads were aligned with the porcine reference genome (Sscrofa 11.1) using the Cell Ranger software (v3.1.0, 10 × Genomics). Subsequent analyses were conducted in Seurat v3(2), applying the following quality-control criteria: 1) Gene count: Cells with fewer than 200 genes or more than 6,500 genes detected were excluded; 2) UMI threshold: Cells with an unusually high number of UMIs (≥50,000) were removed; 3) Mitochondrial content: Cells with >15% of reads mapping to mitochondrial genes were discarded; 4) Multiplets: Potential multiplets from Gel Bead-in-Emulsion (GEM) events were filtered out. The retained high-quality cells were normalized (log normalization), and Harmony(3) was employed to correct for batch effects by grouping cells by cell type. A principal component analysis (PCA) was performed to reduce dimensionality, followed by graph-based clustering in Seurat. The t-SNE was generated using the same principal components for cluster visualization. Cell-type annotation was achieved via SingleR using log-normalized matrices.

To examine how PEDV reshapes the transcriptional program of intestinal goblet cells, we retrieved a publicly available scRNA-seq dataset(4) (GSE175411) from

NCBI-GEO. This dataset comprises jejunal cells from 3-day-old piglets collected at 24 h post-oral challenge with a PEDV variant strain (AH2012/12, GenBank: KU646831, which shares >95% nucleotide identity with the strain used in our study), along with age-matched, uninfected controls(4). We did not generate our own small intestinal data; therefore, the analysis of intestinal goblet cells relied exclusively on this public resource. FASTQ files were processed in Cell Ranger according to the original pipeline, and stringent quality control was performed in Seurat (cells with ≥15% mitochondrial content or fewer than 500 UMIs were excluded). Subsequently, we focused on goblet cell clusters for differential expression and pathway enrichment analyses.

Differential expression, pathway enrichment, and cell communication analysis

A Wilcoxon rank-sum test was used to identify significantly upregulated genes by comparing each cluster against all other cells; genes with an adjusted $p < 0.05$ were considered differentially expressed (DEGs). Enrichment analysis was conducted at the KEGG Pathway level via hypergeometric testing to determine significantly enriched pathways among DEGs relative to the background. clusterProfiler (R) and the annotation package org.Hs.eg.db were used for Gene Ontology (GO) analysis. Intercellular communication was inferred using CellChat, which analyzes ligand–receptor pairs and their expression in each subpopulation, constructs a ligand-receptor-based interaction network, and predicts potential communication events. We compared the interaction strengths under the PEDV vs. Mock and SIV vs. mock conditions. Finally, ggplot2 in R was used to visualize the expression patterns of PEDV- and SIV-related receptors and proteases across all nasal epithelial cell types, SIV-HA and SIV-NP in nasal mucosal cell types, and muscarinic cholinergic receptors in intestinal goblet cells.

Single nuclei RNA sequencing

To investigate how PEDV infection influences the transcriptional landscape of intestinal nerve cells, we established an intestinal ligation model in piglets by

administering PBS or PEDV into the intestinal lumen and collecting jejunal tissue two hours post-inoculation for single-nucleus RNA sequencing (snRNA-seq). Nuclei were isolated using a Nucleus Isolation Kit (SHBIO 52009-10) according to the manufacturer's instructions. RNase inhibitors were added to all reagents to preserve RNA integrity. Freshly dissected intestinal tissue was minced and lysed on ice for three minutes, then passed through a 40-μm filter. Nuclei were counted and assessed for viability using an AO/PI fluorescence-based cell analyzer, followed by 0.4% trypan blue staining under a 40 × microscope to verify nuclear morphology. For library construction, we employed the SeekOne® Digital Droplet Single Cell 3' kit (SeekGene, K00202) and sequenced the resulting libraries on an Illumina NovaSeq 6000 platform with a paired-end 150 bp read configuration. Raw reads were processed using Fastp (v0.20.1) to trim adaptor sequences and remove low-quality bases, and then analyzed using the SeekSoul Tools pipeline to generate a gene expression matrix. Downstream analyses were performed using Seurat v3, including filtration of high- or low-quality nuclei based on gene count, UMI thresholds, and mitochondrial gene proportion. After normalization, PCA was used to reduce dimensionality, and nuclei were clustered using a graph-based approach. Sub-clusters representing neuronal populations were identified, and differential expression analysis (Wilcoxon rank-sum test, adjusted $p < 0.05$) was conducted to pinpoint genes significantly altered by PEDV infection. Finally, GO and KEGG pathway enrichment analyses of these DEGs were performed using ClusterProfiler to elucidate the potential molecular mechanisms underlying PEDV-induced changes in intestinal nerve cells.

Evaluating viral effects on GAP formation and GAP-mediated bacterial translocation in piglet intestines

To investigate whether oral PEDV infection influences the formation of GAPs, 5-day-old piglets were orally inoculated with PEDV (10^5 PFU/mL, 1 mL). After 48 h, the piglets were anesthetized (surgically exposed under isoflurane inhalation anesthesia), and a jejunal loop was created. Then, 200 μL of 10 kDa FITC-Dextran

(2 mg/mL) were introduced into the loop, and after a 45-min incubation, the loop was excised, immediately fixed with 4% paraformaldehyde, embedded in paraffin, sectioned at 4 μm, and examined using laser scanning confocal microscopy to assess GAP formation.

To compare how different viruses affect GAP formation, additional jejunal loops in the same or concurrently treated piglets received one of the following 200 μL treatments 1 h before FITC-Dextran injection: PEDV (10^6 PFU/mL), attenuated PEDV (10^6 PFU/mL), inactivated PEDV (details provided elsewhere), TGEV (10^6 PFU/mL), PDCoV (10^6 PFU/mL), SIV (10^6 PFU/mL), or virus-free control (PBS or medium). One hour after virus inoculation, 200 μL of 10 kDa FITC-Dextran (2 mg/mL) was introduced into the same loop. Following 45-min incubation, the loop was harvested, fixed, embedded, sectioned, and examined by confocal microscopy to evaluate and compare GAP formation across different treatments.

To determine whether PEDV-induced GAPs facilitate bacterial translocation, another set of jejunal loops first received PEDV (10^6 PFU/mL, 200 μL), were incubated for 1 h, and then were given a mixture of 10 kDa FITC-Dextran (2 mg/mL) and mCherry-labeled *E. coli* (10^9 CFU/mL) at a total volume of 200 μL. After a 1-h incubation, the loops were harvested, fixed, embedded, and sectioned. Confocal microscopy was used to assess whether bacteria crossed the intestinal epithelium into the lamina propria. Negative controls included loops not exposed to live viruses and those receiving only the FITC-dextran-bacterial mixture.

Investigation of GAP activation and inhibition in the piglet small intestine

Five-day-old piglets were maintained under standard experimental conditions. Jejunal loops were surgically prepared, and unless otherwise indicated, all treatments were administered intraperitoneally (i.p.). At 30 or 60 min after each intervention, 200 μL of FITC-Dextran (2 mg/mL) was introduced into the loop, followed by a 45-min incubation to evaluate GAP formation via confocal microscopy. To assess ACh-induced GAPs, 0.05 mg/kg ACh was injected 30 min before FITC-dextran, with or

without an additional injection of 0.05 mg/kg AChE to block sustained ACh activity. Submucosal neuronal activation was examined by topically applying 2% lidocaine to the loop surface prior to PEDV inoculation (10^6 PFU/mL). Potential 5-HT involvement was evaluated by administering L-PCPA (75 mg/kg) and ondansetron (0.2 mg/kg) 30 min before PEDV infection. To determine whether CHRM3 mediates PEDV-induced GAPs, 0.05 mg/kg atropine or 0.04 mg/kg 4-DAMP was administered 30 min before PEDV inoculation. Nociceptor engagement was tested by administering AMG517 (1 mg/kg) or A803467 (10 mg/kg) 30 min prior to infection to block TRPV1 and Nav1.8, respectively.

To investigate whether inhibiting GAP formation affects early bacterial translocation during PEDV infection, 0.05 mg/kg 4-DAMP was administered i.p. 12 h before oral PEDV inoculation; at 12 hpi, intestinal tissues were collected for fixed and molecular analyses of mucosal bacterial distribution and epithelial barrier integrity. Furthermore, to assess how GAP inhibition influences PEDV pathogenicity, eighteen newborn piglets of similar weights were randomly allocated to three groups and housed in three separate rooms, the same 4-DAMP regimen (initial injection 12 h pre-infection, followed by 12-h intervals) was maintained. At 48 hpi, half of the piglets were euthanized and tissues were sampled to evaluate the histopathological changes and viral load. The remaining piglets was maintained until 5 dpi, with daily monitoring encompassing body weight measurement, fecal swab sample collection, fecal consistency, diarrhea, appetite, and mental state scoring, and vomiting records, according to established protocols. All procedures were conducted in accordance with the institutional animal welfare guidelines and biosafety regulations, and each group included appropriate replicates for statistical analysis.

In vivo **detection of GAPs in the small intestine of piglets**

To visualize GAPs *in vivo*, 10 kDa FITC-Dextran was administered to the small intestine of anesthetized piglets. After 45 min, the animals were euthanized and the intestinal segments were fixed in 4% paraformaldehyde and subsequently paraffin-embedded. Tissue sections were incubated overnight at 4°C with an anti-Muc2

antibody, followed by the appropriate secondary antibody. Nuclei were counterstained with DAPI. Images were captured using a Zeiss LSM 710 fluorescence microscope and analyzed using ZEN 2012 software (Zeiss). GAPs were defined as Muc2-positive epithelial cells filled with FITC-dextran spanning the epithelium, measuring approximately 20 μm in height and 5 μm in diameter, and containing a nucleus. The number of GAPs was quantified per villus cross-section in the small intestinal tissue.

Histological analysis

Piglets were euthanized, and the nasal and intestinal tissues were collected and fixed with 4% paraformaldehyde for 36 h. After fixation, the tissues were dehydrated in sequential gradient alcohol (75%, 85%, 95%, and 100%) and xylene baths. The tissues were then embedded in paraffin and serially sliced into 5-μm-thick sections. PAS staining was used to determine the mucin content in goblet cells, and hematoxylin-eosin (H&E) staining was used to determine pathological changes. The results were observed using a BX51 digital camera (Olympus Corporation, Tokyo, Japan). Histological lesions of the piglets were quantified as follows. The histology score ranged from 0 to 12 and was subdivided into the following categories: villus architecture (0 = normal to 3 = complete atrophy), epithelial integrity (0 = normal to 3 = extensive shedding or necrosis), inflammatory cell infiltration (0 = none to 3 = severe), and crypt morphology (0 = normal to 3 = marked hyperplasia).

Mucus preparation, total protein extraction, and LC-MS/MS analysis

Jejunal and nasal mucus samples were collected and purified, as previously described(5). Briefly, mucus was gently scraped from the jejunum or nasal cavity of the pigs and suspended in PBS containing a protease inhibitor cocktail (1:100, Sigma Aldrich). After centrifugation at 5,000 × g for 5 min at 4°C to remove insoluble debris, the supernatant was subjected to ammonium sulfate precipitation (final concentration, 45 % w/v) to obtain total mucus proteins. For proteomic analysis, nasal mucus proteins were separated using SDS-PAGE and visualized by Coomassie

Brilliant Blue staining. All visible bands were excised, digested with trypsin, desalted, and analyzed by LC-MS/MS at OE Biotech (Shanghai) on an Ultimate 3000 nano UHPLC coupled to a Q Exactive Plus high-resolution mass spectrometer. To evaluate the integrity of the epithelial barrier during the early phase of PEDV infection (12 h post-inoculation), we profiled the expression of intestinal epithelial junction proteins through LC–LC-MS/MS using the same analytical platform.

Determination of antiviral activity of mucus proteins

Nasal and intestinal mucus proteins were diluted to 500, 50, and 5 μg/ml. Vero E6 cells and MDCK cells were seeded at 2×10^5/well in 12-well cell culture plates and incubated at 37°C until reaching approximately 95% confluence. To verify the virus inactivation ability, mucus proteins were mixed with PEDV or SIV (2×10^4 PFU) individually, and the mixtures were incubated at 37°C for 1 h. The protein mixtures were added to the cells and incubated for 1 h at 37°C, and the culture supernatants were replaced with fresh DMEM and incubated for an additional 24 h. To assess the ability to resist virus invasion, mucus proteins were added to Ver E6 or MDCK cells and incubated at 37°C for 30 min. Subsequently, the cells were inoculated with 0.1 MOI of either PEDV or SIV and incubated at 37°C for 1 h. After discarding the liquid, the cells were washed with sterile PBS and then incubated in fresh DMEM at 37°C for 24 h. The cell samples were assessed through western blotting using specific antibodies. At the indicated time points post infection, the collected cell samples were lysed in RIPA buffer containing a protease inhibitor cocktail (Thermo Fisher Scientific). The lysate concentration was determined using a BCA protein quantification kit (Thermo Fisher Scientific). Equal amounts of proteins were separated using SDS-PAGE and electrophoretically transferred onto polyvinylidene fluoride membranes (Millipore, Shanghai, China). The membranes were blocked with 5% skim milk for 2 h and incubated with specific primary antibodies, followed by incubation with appropriate horseradish peroxidase-conjugated secondary antibodies. The intensity of the bands in terms of density was measured and normalized to that of glyceraldehyde 3-phosphate dehydrogenase

(GAPDH).

Quantitative reverse-transcription PCR (RT-qPCR)

Quantitative viral loads and host genes were determined using real-time quantitative PCR. Total RNA was extracted from tissues and cells according to the TRIzol total RNA extraction protocol (Invitrogen). Reverse transcription was performed using the HiScript III RT SuperMix (Vazyme, Nanjing, China). RT-qPCR was performed using the ChamQ Universal SYBR qPCR Master Mix (Vazyme) on a 7500 Fast Real-Time PCR System (Applied Biosystems, Foster City, CA, USA). GAPDH was used as an internal control to quantify the amount of cDNA. Relative expression levels of RNA were calculated using the $2^{-\Delta\Delta CT}$ method. Primers specific for each virus and host gene are listed in Table S1.

Immunohistochemistry (IHC) and indirect immunofluorescence assay (IFA)

Nasal and intestinal tissues were immersed in 4% paraformaldehyde for 36 h before being embedded in paraffin wax. Paraffin-embedded sections were sectioned serially at a thickness of 5 μm using a microtome (Leica RM2235). The paraffin-embedded sections were dewaxed in xylene and rehydrated using decreasing concentrations of ethanol. Antigen retrieval was performed for 20 min with EDTA antigen retrieval solution in a Decloaking Chamber at 95 °C. For IHC analysis, slides were incubated with anti-PEDV N or anti-SIV HA antibody overnight at 4°C in a humidified chamber. The SABC-POD Kit was used for signal amplification and visualization. The numbers of $PEDV^+$ and SIV^+ cells in the nasal and jejunal of each piglet were counted for statistical analysis. For IFA, sections were incubated with primary antibodies toward PEDV N protein, Muc2, MUC5AC, CHGA, serotonin, LAMP1, Rab7a, TGN46, and pEGFR at 4°C overnight, then incubated with goat anti rabbit or goat anti mouse Alexa Fluor conjugated secondary antibodies. Finally, the sections were stained with DAPI and visualized by LSM 710 immunofluorescence microscope (Zeiss). Images were analyzed using the ZEN 2012 software (Zeiss). The mean fluorescence intensity was calculated for statistical analysis.

Fluorescence in-situ hybridization (FISH)

The FISH experiment was performed according to the SweAMI-FISH manufacturer's instructions (Servicebio). Tissue sections were dewaxed, and antigens were retrieved using EDTA antigen retrieval solution. Then, the sample was digested using proteinase K (20 μg/ml) at 40°C for 5 min and washed. The sample was treated using the prehybridization solution at 37°C for 1 h, then treated using the hybridization solution (containing 500 nM FISH-probe) at 40°C overnight. Subsequently, SSC solutions (2 ×, 1 ×, and 0.5 ×) of different concentrations were used to wash the sample. The sample was incubated with the signal probe (diluted with hybridization solution) at 40°C for 1 h, followed by washing. Finally, the sections were stained with DAPI and the fluorescence images were captured using a laser confocal microscope (Zeiss). The sequences of the fluorescent in situ hybridization probes are listed in Table S1.

ELISA

To investigate the effect of PEDV infection on the production of neurotransmitters and inflammatory factors, intestinal tissue samples were collected from piglets. Acetylcholine and choline acetyltransferase assay kits (Jiancheng Bioengineering Institute, Nanjing, China) were used to detect ACh concentration and ChAT activity, respectively. 5-HT, IL-1β, and lipopolysaccharide ELISA kit (mlbio, Shanghai) were used to detect the 5-HT, IL-1β, and lipopolysaccharide concentration according to manufacturer's instructions, respectively. Absorbance (OD450 nm) was measured using a Tecan Spark multifunctional microplate reader.

Quantification and statistical analysis

All data are presented as mean ± standard deviation (SD). Analyses were performed with SPSS v17.0 (SPSS Inc., Chicago, IL, USA). For comparisons involving more than two groups, a one-way ANOVA was first conducted; when the overall F-test was significant ($P < 0.05$), pair-wise differences were evaluated with Fisher's least-significant-difference (LSD) post-hoc test. When only two groups were compared,

an unpaired two-tailed Student's t-test was used. Significance was set as follows: *, $p < 0.05$; **, $p < 0.01$; and ***, $p < 0.001$. Data were combined from at least three independent experiments unless otherwise stated.

References

1. Li Y*, et al.* (2018) An alternative pathway of enteric PEDV dissemination from nasal cavity to intestinal mucosa in swine. *Nature communications* 9(1):3811.
2. Stuart T*, et al.* (2019) Comprehensive Integration of Single-Cell Data. *Cell* 177(7):1888-1902.e1821.
3. Korsunsky I*, et al.* (2019) Fast, sensitive and accurate integration of single-cell data with Harmony. *Nature methods* 16(12):1289-1296.
4. Fan B*, et al.* (2023) Identification of Cell Types and Transcriptome Landscapes of Porcine Epidemic Diarrhea Virus-Infected Porcine Small Intestine Using Single-Cell RNA Sequencing. *Journal of immunology (Baltimore, Md. : 1950)* 210(3):271-282.
5. Li Y*, et al.* (2022) Calpain-1: a Novel Antiviral Host Factor Identified in Porcine Small Intestinal Mucus. *mBio* 13(5):e0035822.

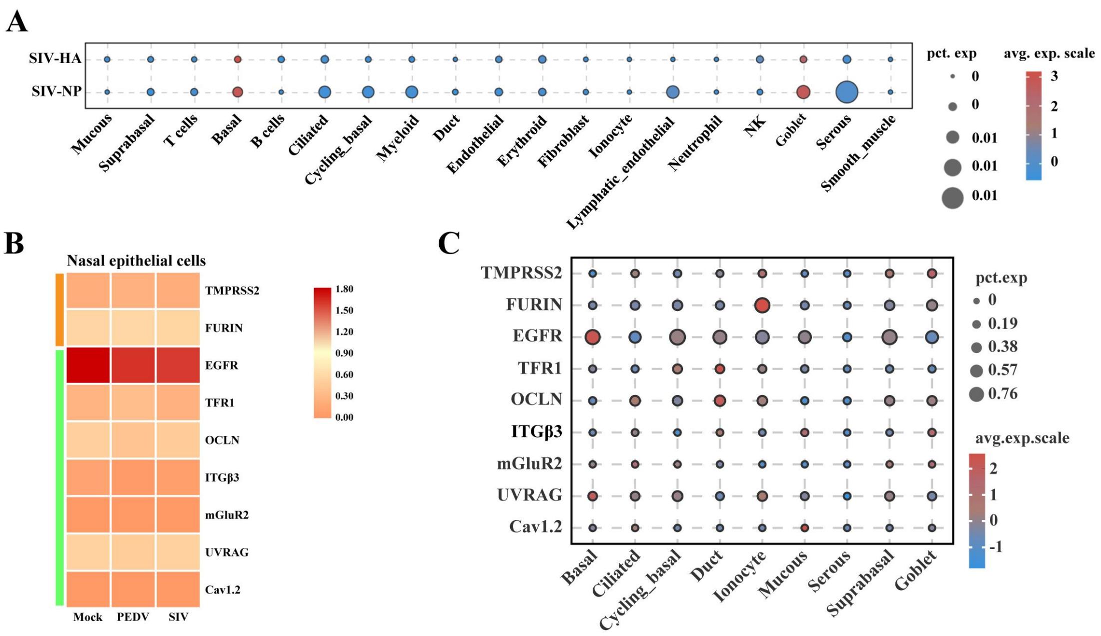

Figure S1. Impact of PEDV and SIV on nasal mucosal cell composition and the distribution of SIV transcripts, virus receptors, and host proteases in nasal epithelial cells.

(A) Transcriptional profiles of SIV-related genes in nasal mucosal cells. (B) Transcriptional profiles of proteases (orange module) and receptors (green module) involved in PEDV and SIV invasion and infection in nasal epithelial cells. (C) Transcriptional profiles of receptors and proteases involved in PEDV and SIV invasion and infection in nasal epithelial cell subsets.

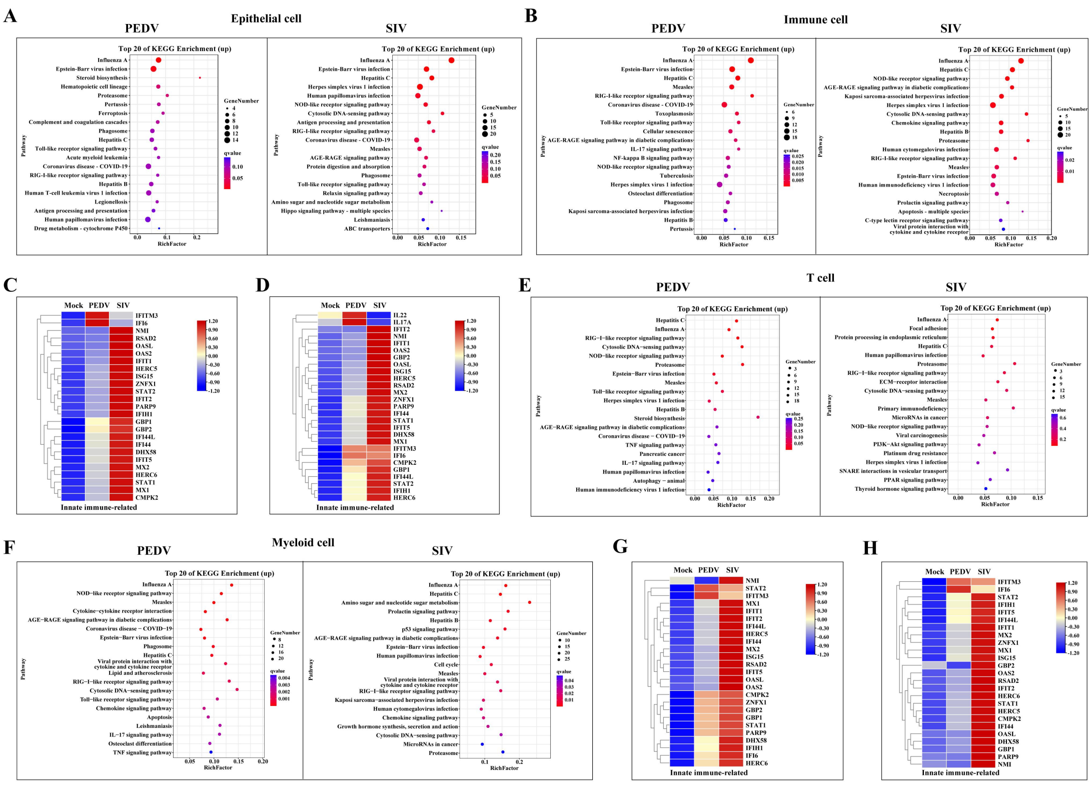

Figure S2. Transcriptional characteristics of nasal epithelial and immune cells in response to PEDV or SIV infection.

(A and B) KEGG enrichment analysis of differentially expressed genes in nasal epithelial (A) and immune cells (B) after PEDV or SIV infection. (C and D) Transcriptional profiles of innate immune-related genes in nasal epithelial (C) and immune (D) cells. (E and F) KEGG enrichment analysis of differentially expressed genes in T (E) and myeloid (F) cells after viral infection. (G and H) Transcriptional profiles of innate immune-related genes in T (G) and myeloid (H) cells.

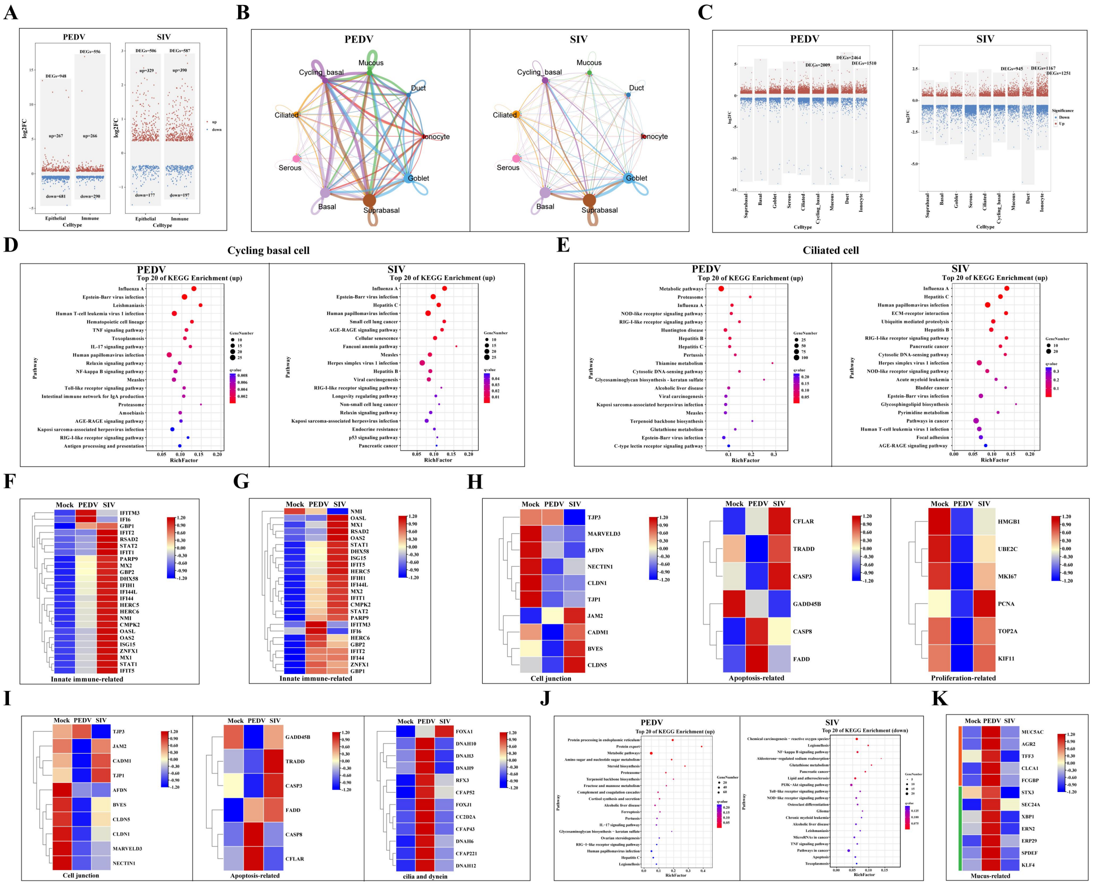

Figure S3. Differential responses of nasal epithelial cell subsets infected with PEDV or SIV.

(A) Volcano plots showing differentially expressed genes in nasal epithelial and immune cells. (B) Predicted interaction strength among epithelial cell subpopulations in the PEDV or SIV infection groups. (C) Volcano plot illustrating differentially expressed genes across nasal epithelial cell subpopulations. (D and E) KEGG enrichment analysis of differentially expressed genes in nasal cycling basal cells (D) and ciliated cells (E) after PEDV or SIV infection. (F and G) Transcriptional profiles of innate immune-related genes in cycling basal (F) and ciliated (G) cells. (H) Transcriptional profiles of intercellular junction molecules, apoptosis-related genes, and cell proliferation-related genes in cycling basal cells. (I) Transcriptional profiles of intercellular junction molecules, apoptosis-related genes, and cilium formation and motility-related genes in ciliated cells. (J) KEGG enrichment analysis of differentially expressed genes in goblet cells after PEDV or SIV infection. (K) Transcriptional profiles of mucin-related (orange module) and

mucus secretion-related (green module) genes in goblet cells.

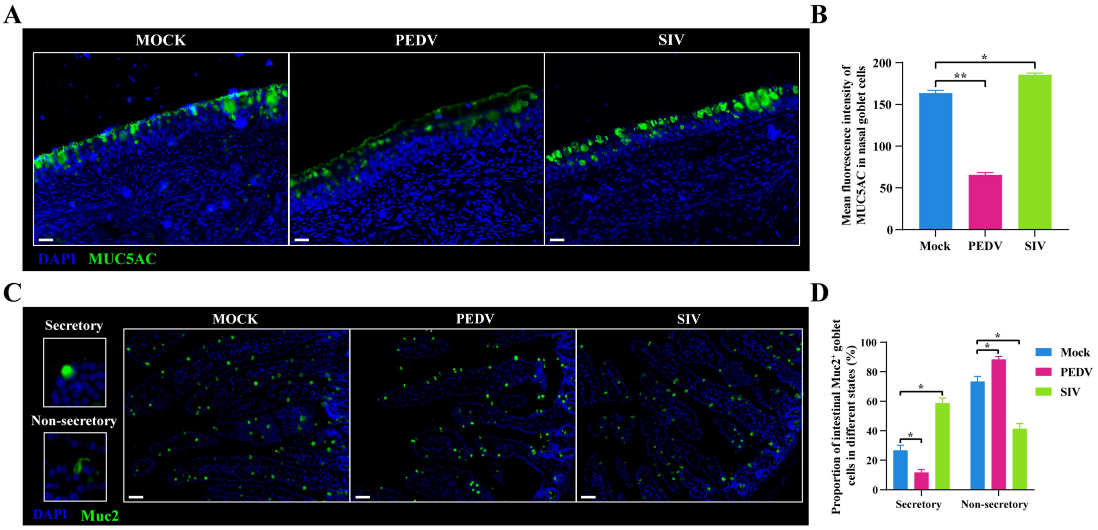

Figure S4. PEDV and SIV differentially affect signature mucin secretion by nasal and intestinal goblet cells.

(A) Representative confocal images showing MUC5AC (green) distribution in nasal mucosa of piglets intranasally inoculated with PEDV or SIV. Nuclei: DAPI (blue). Scale bar: 20 μm. (B) Quantification of intracellular MUC5AC fluorescence intensity in goblet cells from (A). Data from five random goblet cells per section, three sections per group. (C) Representative confocal images showing Muc2 (green) distribution in intestinal mucosa of piglets orally inoculated with PEDV or SIV, highlighting secretory versus non-secretory goblet cells. Nuclei: DAPI (blue). Scale bar: 50 μm. (D) Proportion of secretory vs. non-secretory goblet cells quantified from (C). Data from four random microscopic fields per group. Data represent the mean results ± SD from three independent experiments. * $p < 0.05$, ** $p < 0.01$. The error bars indicate the range.

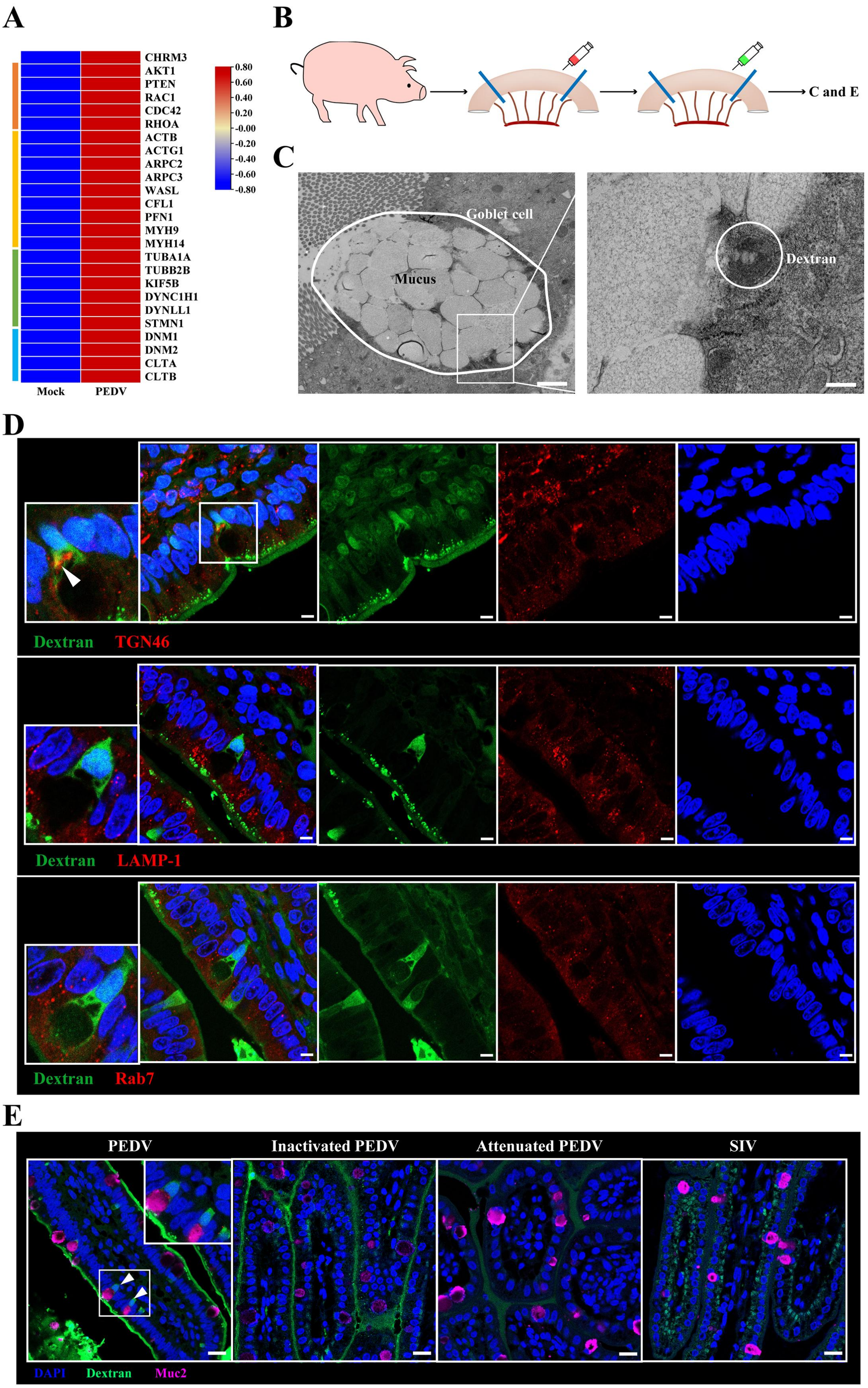

Figure S5. Specific GAP formation by PEDV characterized by transcriptional,

structural and functional features.

(A) Transcriptional profiles of genes associated with GAP formation in small intestinal goblet cells, categorized into PI3K-AKT signaling (orange), actin polymerization (yellow), microtubule transport (green), and endocytosis (blue) modules. (B) Schematic of the piglet intestinal ligation model established to assess GAP formation in the small intestine following PEDV inoculation. (C) Transmission electron microscopy images of GAP formation in goblet cells at 2 h post-PEDV infection, showing dextran-containing structures; scale bars: 1 μm (left), 200 nm (right). (D) Confocal microscopy showing colocalization of GAPs (FITC-dextran, green) with TGN46 (red), LAMP-1 (red), and Rab7 (red) in the piglet small intestine. Nuclei were stained with DAPI (blue). Scale bar: 5 μm. (E) Confocal microscopy of GAP formation (green) in goblet cells (pink) 2 h post-infection with PEDV, inactivated PEDV, attenuated PEDV, or SIV; nuclei stained with DAPI (blue). Scale bar: 20 μm.

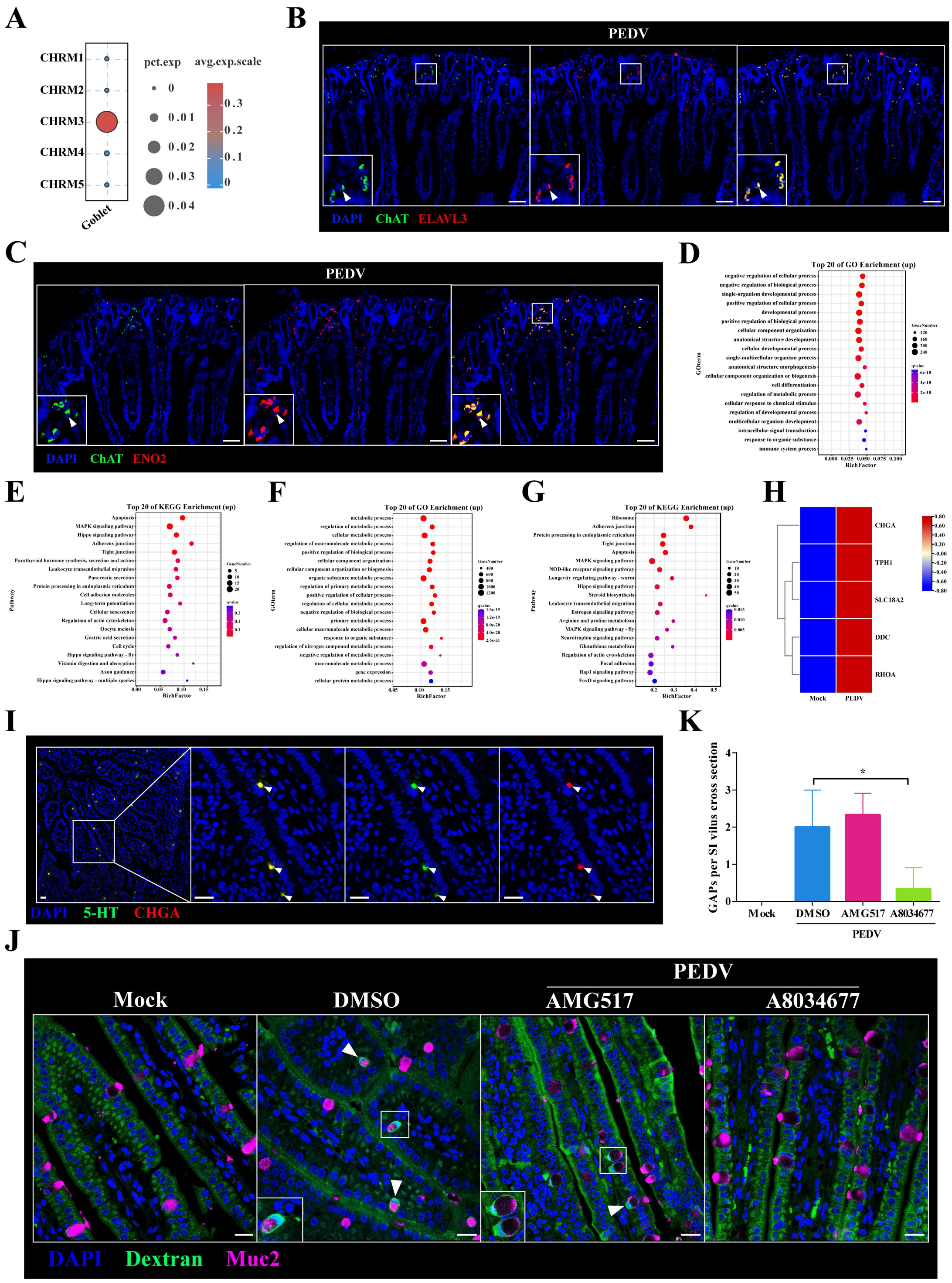

Figure S6. GAP formation is not related to tuft cells but is associated with nav1.8 activation.

(A) Transcriptional profiling of muscarinic cholinergic receptors in intestinal goblet cells. (B and C) Fluorescent probes targeting neural cell markers ELAVL3 and ENO2 were designed. Confocal microscopy was used to examine the colocalization

of ChAT (green) with ELAVL3 (red) or ENO2 (red) in piglet small intestine tissue. Nuclei were stained with DAPI (blue). Scale bar: 20 μm. (D and E) GO (D) and KEGG (E) enrichment analyses of differentially expressed genes in tuft cells. (F and G) GO (F) and KEGG (G) enrichment analyses of differentially expressed genes in enteroendocrine cells. (H) Transcriptional profiles of genes involved in 5-HT synthesis in enteroendocrine cells. (I) Confocal microscopy showing the colocalization of 5-HT (green) and enteroendocrine cells (CHGA, red) in piglet small intestine tissue post-PEDV infection. Nuclei were stained with DAPI (blue). Scale bar: 20 μm. (J) Confocal microscopy showing GAP formation (FITC-dextran, green) within goblet cells (pink) of the piglet small intestine under various treatment conditions. Nuclei were stained with DAPI (blue). Scale bar: 20 μm. (K) Quantitative assessment of GAPs per cross-section of intestinal villi shown in panels (J). Data were obtained from three sections, with 15 randomly selected villi per section. Data represent the mean results ± SD from three independent experiments. * $p < 0.05$. The error bars indicate the range.

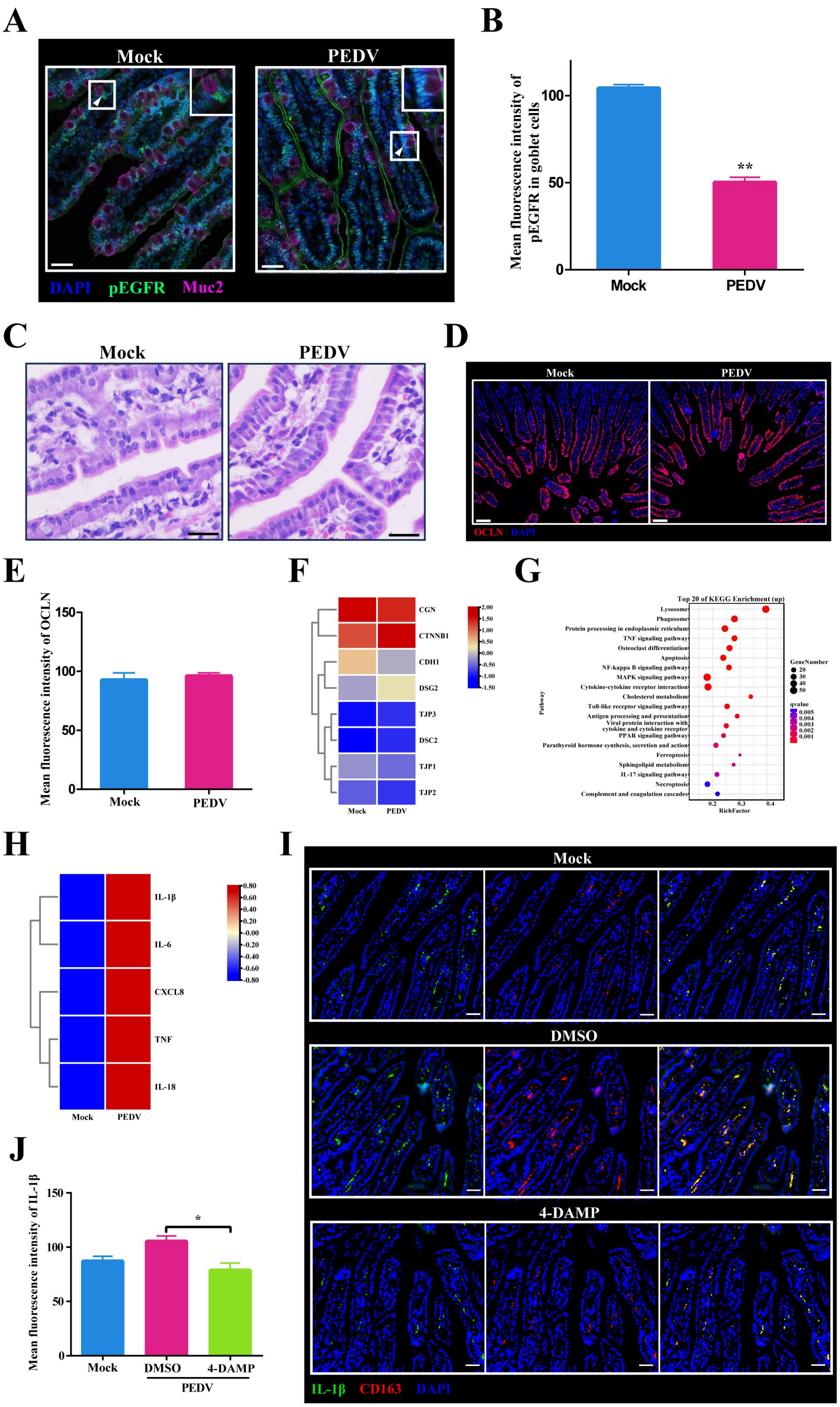
A
Mock
PEDV
DAPI pEGFR Muc2
B
Mean fluorescence intensity of pEGFR in goblet cells
100
50
0
**
Mock
PEDV
C
Mock
PEDV
D
Mock
PEDV
OCLN DAPI
E
Mean fluorescence intensity of OCLN
150
100
50
0
Mock
PEDV
F
CGN
CTNNB1
CDH1
DSG2
TJP3
DSC2
TJP1
TJP2
Mock
PEDV
2.00
1.50
1.00
0.50
0.00
-0.50
-1.00
-1.50
G
Top 20 of KEGG Enrichment (up)
Lysosome
Phagosome
Protein processing in endoplasmic reticulum
TNF signaling pathway
Osteoclast differentiation
Apoptosis
NF-kappa B signaling pathway
MAPK signaling pathway
Cytokine-cytokine receptor interaction
Cholesterol metabolism
Toll-like receptor signaling pathway
Antigen processing and presentation
Viral protein interaction with cytokine and cytokine receptor
PPAR signaling pathway
Parathyroid hormone synthesis, secretion and action
Ferroptosis
Sphingolipid metabolism
IL-17 signaling pathway
Necroptosis
Complement and coagulation cascades
Pathway
RichFactor
GeneNumber
20
30
40
50
qvalue
0.005
0.004
0.003
0.002
0.001
H
IL-1β
IL-6
CXCL8
TNF
IL-18
Mock
PEDV
0.80
0.60
0.40
0.20
-0.00
-0.20
-0.40
-0.60
-0.80
I
Mock
DMSO
4-DAMP
IL-1β CD163 DAPI
J
Mean fluorescence intensity of IL-1β
150
100
50
0
*
Mock
DMSO
4-DAMP
PEDV

Figure S7. GAPs induced by PEDV promote intestinal inflammation.

(A) Confocal microscopy showing phosphorylated EGFR (green) expression in goblet cells (Muc2, pink) of the piglet small intestine. Nuclei were stained with DAPI (blue). Scale bar: 40 μm. (B) Quantification of phosphorylated EGFR fluorescence intensity shown in panel (A). Values were derived from three sections, with five randomly selected goblet cells per section. (C) H&E staining assessed the impact of PEDV infection (12 h) on small intestinal epithelial integrity. Scale bar: 20 μm. (D) Confocal microscopy showing expression of the tight junction protein Occludin (red) in the piglet small intestinal mucosa. Nuclei were stained with DAPI (blue). Scale bar: 100 μm. (E) Quantification of OCLN fluorescence intensity shown in panel (D). Values were derived from three sections, with 15 randomly selected villi per section. (F) Expression profiles of intercellular junction protein in small intestinal tissues. (G) KEGG pathway enrichment analysis of differentially expressed genes in intestinal macrophages. (H) Transcriptional profiles of inflammation-related genes in intestinal macrophages. (I) Confocal microscopy showing colocalization of IL-1β (green) and the macrophage marker CD163 (red) in the piglet small intestinal mucosa using fluorescent probes. Nuclei were stained with DAPI (blue). Scale bar: 50 μm. (J) Quantification of IL-1β fluorescence intensity shown in panel (I). Values were derived from three sections, with 15 randomly selected villi per section. Data represent the mean results ± SD from three independent experiments. * $p < 0.05$, ** $p < 0.01$. The error bars indicate the range.

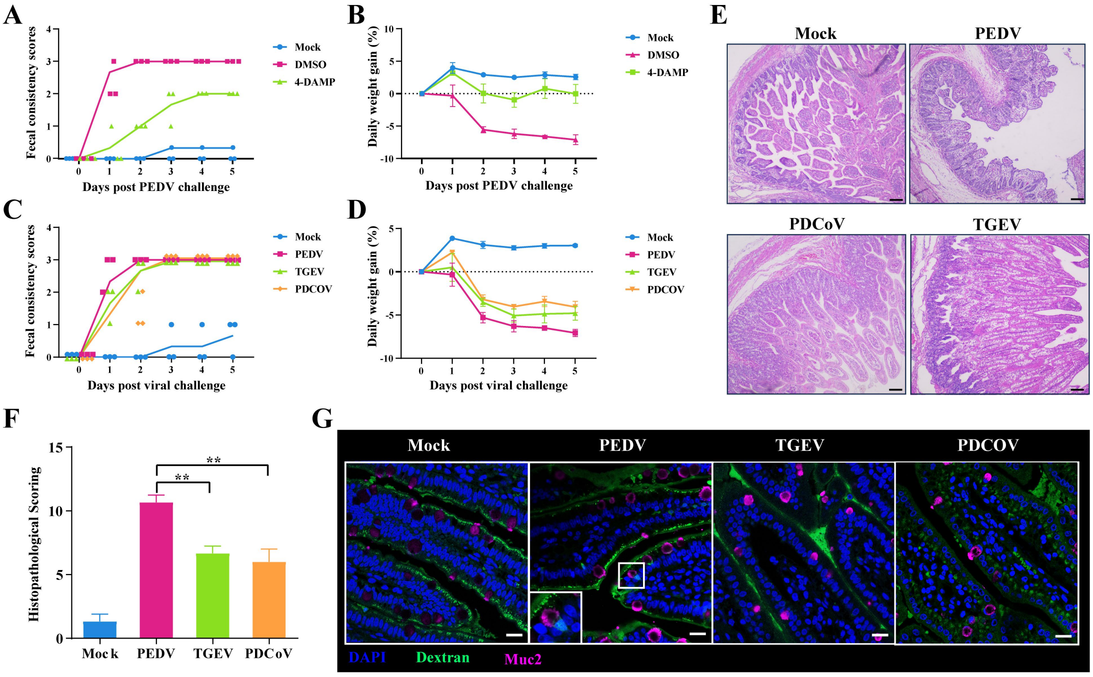

Figure S8. GAP formation induced by PEDV is an important factor contributing to its high pathogenicity.

(A and B) Supplementary data for the *in vivo* challenge study examining the role of GAP induction in PEDV intestinal infection and pathogenicity. (A) Diarrhea was assessed by scoring fecal consistency (0, solid; 1, pasty; 2, semiliquid; and 3, liquid). A score ≥2 was considered diarrhea. (B) Average daily weight gain rate of piglets from each group. (C-G) *In vivo* challenge study designed to compare and characterize the pathogenic features of different enteric coronaviruses. (C) Diarrhea was assessed by scoring fecal consistency (0, solid; 1, pasty; 2, semiliquid; and 3, liquid). A score ≥2 was considered diarrhea. (D) Average daily weight gain rate of piglets from each group. (E) Hematoxylin and eosin (H&E) staining of the piglet jejunum post-infection with PEDV, TGEV, or PDCoV. Scale bar: 100 μm. (F) Histological scoring of the piglet jejunum infected with PEDV, TGEV, or PDCoV. (G) Confocal microscopy showing GAP formation (FITC-dextran, green) in goblet cells (pink) of the piglet small intestine under different treatment conditions. Nuclei were stained with DAPI (blue). Scale bar: 20 μm. Data represent the mean results ± SD from three independent experiments. ** $p < 0.01$. The error bars indicate the range.

Table S1. Prime and probe sequences used for RT-qPCR and FISH detection

Gene	Primers	Sequence (5'-3')
**For RT-qPCR detection**		
GAPDH	Forward	TCGGAGTGAACGGATTTGGC
	Reverse	TGACAAGCTTCCCGTTCTCC
PEDV-N	Forward	GCAAAGACTGAACCCACTAAC
	Reverse	CATTACCACGACTCCTGCT
SIV-HA	Forward	ATAGCAGCAAGACCCAAGG
	Reverse	CGTGGACTGGTGTATCTGAA
CLCA1	Forward	CAAGTGTGTTTCAGCCGA
	Reverse	AGGTTGATGAGGTTGTCCC
FCGBP	Forward	GGGAACGAGAGCATTCTTT
	Reverse	GCCCAAGGAACAGGTATCT
MUC5AC	Forward	TGAGAGCCAAGGGATACAG
	Reverse	CTGGTCTTTGTTCAGGCAA
AGR2	Forward	ATTGGCAGAGCAGTTCGT
	Reverse	CGGAAGGTTCATACGCAT
ChAT	Forward	CAATCGGGACTCTCTGGATA
	Reverse	CAAACTGTAGGGACTTGTCGTA
TLR2	Forward	GCCAATAGCATTCATACCG
	Reverse	TTCAAGGTGGAAAGGGACT
**For FISH detection**		
ChAT	Probe	AAACTGCTGCACAATGGCCTGGCTCCTCCT AAATTG-CY5
ELAVL3	Probe	TTGCTGTCATCAGTGGCTCCGTTTGTCCCAA GGAGT-CY3
ENO2	Probe	TTGCCTAAGTATCGCTGTTTGTCACCATCCC TTAGC-CY3
EUB338	Probe	GCTGCCTCCCGTAGGAGT-CY3
IL-1β	Probe	TTTCTCAGAGAACCAAGGTCCAGG-FITC
CD163	Probe	GCAGACCTGAAACTGAGAGTGGTA-CY3

Table S2. Clinical symptom scores of piglets after PEDV challenge

Group	dpi	Diarrhea[a]	Appetite[b]	Mental state[c]	Vomiting[d]
	1 d	0/0/0	0/0/0	0/0/0	0/0/0
	2 d	0/0/0	0/0/0	0/0/0	0/0/0
Mock	3 d	0/0/0	0/0/0	0/0/0	0/0/0
	4 d	1/0/0	1/0/0	0/0/0	0/0/0
	5 d	0/1/0	0/0/0	0/0/0	0/0/0
	1 d	2/2/3	0/1/1	0/0/1	0/0/0
	2 d	3/3/3	1/1/2	1/1/1	1/1/1
DMSO	3 d	3/3/3	2/2/2	2/2/2	2/2/2
	4 d	3/3/3	2/2/2	2/2/2	2/2/2
	5 d	3/3/3	2/2/2	2/2/2	1/1/1
	1 d	0/0/0	0/0/0	0/0/0	0/0/0
	2 d	1/1/1	0/0/0	0/0/0	0/0/0
4-DAMP	3 d	1/2/2	1/1/1	0/1/1	0/1/1
	4 d	2/2/2	1/1/1	1/1/1	0/0/1
	5 d	2/2/2	1/1/1	1/1/1	0/0/0

[a] Diarrhea: 0 = normal; 1 = soft stools; 2 = mild diarrhea; 3 = watery/mucoid stools (bloody rare).

[b] Appetite: 0 normal; 1 intake < 50 % of normal; 2 ≥ 50 % reduction or refusal.

[c] Mental state: 0 alert; 1 slightly lethargic; 2 recumbent/unresponsive.

[d] Vomiting: 0 none; 1 one episode/day; 2 ≥ two episodes/day.

Scores are listed as “pig 1 / pig 2 / pig 3”; dpi = days post-infection. Mock = uninfected control; DMSO = vehicle-treated PEDV group; 4-DAMP = GAP-inhibited PEDV group.
